# Thymic Microenvironment Remodeling in Cancer Cachexia as a Determinant of Checkpoint Inhibitor Efficacy and Toxicity

**DOI:** 10.1002/jcsm.13874

**Published:** 2025-07-16

**Authors:** Run‐Kai Huang, Yan‐Fang Xing, Xiang‐Yuan Wu, Zhao‐Hui Shi, Li Wei, Xiao‐Tong Lv, Lin‐Jiao Peng, Xiu‐Qing Pang, Qin‐Tai Yang, Xing Li

**Affiliations:** ^1^ Department of Medical Oncology The Third Affiliated Hospital of Sun Yat‐sen University Guangzhou China; ^2^ Guangdong Key Laboratory of Liver Disease Research The Third Affiliated Hospital of Sun Yat‐sen University Guangzhou China; ^3^ Department of Neurology The Third Affiliated Hospital of Guangzhou Medical University Guangzhou China; ^4^ Department of Allergy and Department of Otorhinolaryngology‐Head and Neck Surgery The Third Affiliated Hospital of Sun Yat‐sen University Guangzhou China; ^5^ Department of Infectious Diseases The Third Affiliated Hospital of Sun Yat‐sen University Guangzhou China

**Keywords:** cancer cachexia, immune checkpoint inhibitors, T cell negative selection, thymus involution, thymus medullary fibroblast

## Abstract

**Background:**

The discovery of immune checkpoints links autoimmunity and cancer, with thymus atrophy reportedly causing autoimmune multiorgan inflammation. The impact of cancer cachexia on thymic involution and its clinical significance remains unclear. This study aimed to investigate this effect and its association with immune checkpoint inhibitor (ICI) treatment.

**Methods:**

Single‐cell sequencing, immunofluorescence and flow cytometry analyses were conducted to explore changes in the thymus in orthotopic hepatocellular cancer (HCC) mice with cachexia. Patients with advanced and locally advanced cancers receiving anti‐PD‐1/L1 antibody treatment were followed up to investigate the relationship between the amount of serum autoantibodies and the efficacy of ICIs.

**Results:**

Single‐cell sequencing in cachexic HCC mice revealed thymic fibroblast maturity disorders characterized by elevated immature medullary fibroblasts, impaired antigen processing functions, reduced interaction with single‐positive thymocytes and decreased expression of tissue‐restricted antigen‐related genes. The thymus of mice with cancer cachexia exhibited degradation of the thymic medulla and decreased expression of LtβR, Mmp9 and Ccl19 in thymus medullary fibroblasts (mFbs). Single‐cell TCR sequencing showed that inflammatory‐related V/J TCR genes were highly used in expanded thymocyte clonotypes in cachexic HCC mice, suggesting impaired T cell negative selection. Results from coculture and cell transfer assays suggest that cancer cachexic CD45^+^ erythroid progenitor cells (EPCs) induce the death of CD34^+^ progenitor cells and decrease the number of LtβR^+^, Mmp9^+^ and Ccl19^+^ mFbs in tumour‐free mice. CD24^+^CD4^+^CD8^−^ single‐positive thymocytes, typically eliminated in negative selection, did not decrease after the administration of anti‐CD3 mAb. Serum autoantibodies were markedly produced in cachexic HCC mice, cachexic HCC mice administered with anti‐PD1 and tumour‐free mice that received cancer cachexic CD45^+^ EPCs. Autoantibodies against tumour‐restricted antigens were found in patients with advanced and locally advanced cancer who received two cycles of ICI treatment. Univariate Cox regression analysis showed that patients with a low level of autoantibodies had a higher risk of disease progression (hazard ratio [HR]: 2.39, 95% CI [1.02–5.63], *p* = 0.046). Analysis of the receiver operating characteristic curve indicated that the number of autoantibodies against tumour tissues predicted treatment failure (area under the curve [AUC] 0.726, *p* = 0.021) and long‐term duration of treatment response (AUC 0.697, *p* = 0.024). Patients with high levels of serum autoantibodies against tumours had favourable progression‐free survival (HR, 0.389; 95% CI [0.158–0.960], *p* = 0.04).

**Conclusions:**

Cancer cachexia disrupts mFbs maturity, affecting T cell negative selection and expanding the TCR repertoire against tissue‐restricted antigens. This might mediate the adverse and favourable effects of ICIs as anticancer treatments.

## 1 Introduction

The discovery of immune checkpoints and their inhibitors (ICIs) suggests a close association between autoimmunity and cancer. Tumour‐associated antigens (TAAs) and tumour‐specific antigens (TSAs) are recognized by T cells and trigger an anticancer immune response [1]. T cells that respond to TAAs, including cancer germline antigens, are eliminated by central tolerance, leading to cancer immune tolerance and the prevention of immune‐related adverse events (irAEs) against these antigens [1]. However, thymus atrophy develops owing to multiple causes, including age, chemotherapy and sepsis [2]. Developmental disorders of thymocytes and impaired T cell negative selection during thymus atrophy may influence the efficacy of ICIs. Immune checkpoints maintain peripheral tolerance by preventing autoimmune responses against tissue‐restricted antigens (TRAs) and limiting adaptive immunity targeting TAAs.

ICIs for treating multiple cancers activate the immune system against TAAs; however, their effectiveness is accompanied by unpredictable irAEs [3]. Disruption of peripheral tolerance through ICIs triggers irAEs, which can also manifest when central tolerance becomes dysfunctional [4]. Medullary thymic epithelial cells (mTECs), fibroblasts and regulatory T cells (Tregs) are reportedly involved in T cell negative selection through TRAs expression [5]. Thymus atrophy reportedly impairs T cell negative selection, leading to autoimmune inflammation in multiple organs, which might also be induced by cancer attacks [2]. Malignancies cause cell differentiation and dysplasia in multiple cell types in distant organs, mainly in haematopoietic cells. Tumours cause the accumulation and activation of myeloid‐derived suppressor cells (MDSCs) [6], erythroid progenitor cells (EPCs) [7] and erythroid‐transdifferentiated myeloid cells [8]. MDSCs and EPCs affect the normal functioning of multiple cells, including endothelial cells, via circulation [9, 10]. Thus, thymus involution might occur in patients at certain stages of cancer and lead to impaired T cell negative selection, which ultimately mediates the favourable and adverse effects of ICIs.

Cachexia is prevalent among patients with cancers and is characterized by malnutrition, which induces thymus involution, as evidenced by severe atrophy and an impaired thymic microenvironment [11]. However, pathological changes in the thymus during cancer cachexia and their clinical importance remain unclear. This study aimed to conduct single‐cell mapping of the thymus using hepatocellular carcinoma (HCC) in situ mouse models with cachexia and investigate the causal association between thymus dysfunction and ICI effects, as well as their potent mechanisms and clinical applications.

## 2 Methods

### 2.1 Mice

C57BL/6 mice (male, 6 weeks old) were purchased from Guang Dong Medical Laboratory Animal Center, and rag1^−/−^ mice (male, 6 weeks old) were purchased from Shanghai Model Organisms Center Inc. All mice were housed under pathogen‐free conditions and all animal experiments were performed with the approval of the Institutional Animal Care and Use Committee of the Third Affiliated Hospital of Sun Yat‐sen University.

### 2.2 Orthotopic HCC Mouse Model

Hepa1‐6 cells at the logarithmic growth stage were digested with pancreatic enzymes and prepared as a mixture of 1 × PBS and Matrigel matrix glue (Corning, NY, USA) at a 1:1 ratio for a concentration of 1 × 10^6^ cells per 20 μL. After anaesthetizing the mice with 160 μL of 1% pentobarbital per 25 g of body weight, they were sagittally incised in the middle of the abdomen. Next, the median liver lobe was exteriorized and gently placed onto the peritoneum. A fine‐gauge needle was then inserted, and 20 μL of a *Hepa1‐6* cell/Matrigel suspension was injected into the subcapsular region of the lobe. The peritoneum and skin were subsequently closed using interrupted sutures with 5‐0 suture material. Finally, the mice were maintained on a thermal blanket until fully recovered from anaesthesia.

### 2.3 CD45^+^ EPC Transfer Experiment

CD45^+^ cells were enriched by magnetic activated cell sorting (MACS) in the spleen and circulation of cachexic HCC mice. The cells were incubated with CD45^+^biotin (Cat#13‐0451‐82, Invitrogen, Waltham, Massachusetts, USA) for 30 min. After washing with 1 × PBS, the cells were incubated with anti‐biotin beads (MSPB‐6003‐74; Invitrogen) for 30 min. CD45^+^ cells were harvested using a magnetic rack. The cells were stained with ter119 (Cat#50‐5921‐U100, TONBO Biosciences) and CD71 (Cat#17–0711‐82, Invitrogen) to collect CD45^+^ EPCs using fluorescence‐activated cell sorting (FACS). Next, 1.5 × 10^6^ CD45^+^ EPC from the spleen and circulation of cachexic HCC mice were transferred into tumour‐free adult mice via tail vein injection, twice a week for 4 weeks. Thymi were isolated for analysis of LtβR^+^ mFb, Mmp9^+^ mFb and Ccl19^+^ mFb using flow cytometry or immunofluorescence.

### 2.4 Coculture Assay

To explore whether CD45^+^ EPCs impair the viability of CD34^+^ progenitor cells, we established a transwell coculture system. CD45^+^ EPCs from the spleen and circulation of cachexic HCC mice, as well as from patients with cancer cachexia, were collected using MACS and FACS. CD34^+^ progenitor cells were isolated from the bone marrow of cachexia HCC mice and cord blood of human. CD34^+^ progenitor cells were seeded in the bottom chamber; CD45^+^ EPCs were incubated in the upper chamber at 1:0, 1:1, 1:2 and 1:4 ratios overnight. CD34^+^ progenitor cell viability was assessed using trypan blue.

## 3 Results

### 3.1 Thymic Fibroblasts in Mice With Cachexic Tumours Displayed a Maturity Disorder

Single‐cell RNA sequencing was performed on total thymic cells from cachectic HCC mice, defined by a 5%–10% loss in body weight, skeletal muscle mass and fat (serving as the humane endpoint) (*n* = 2), and sham‐operated control mice (*n* = 2), to investigate the impact of cancer cachexia on the adult mouse thymuses (Figure S1). After quality control, 23 813 and 23 261 cells were retained from sham and cachectic HCC mice, respectively (Figure S2A). Using previously reported marker genes (S1–S17) (Figure S2B, Figure S3A and Table S1), we performed clustering analysis combined with two‐dimensional projection to examine the composition of thymic cell populations, including double‐negative (DN) thymocytes, double‐positive (DP) blasts (DPblasts), DP thymocytes undergoing rearrangement (DPres), DP thymocytes undergoing selection (DPsels), CD4/CD8 single‐positive (SP) thymocytes, natural killer T (NKT) cells, Tregs, γδ T cells, B cells, conventional dendritic cells (cDCs), plasmacytoid dendritic cells (pDCs) and migratory dendritic cells (Figure S2A). Overall, the cellular compositions of the thymus appeared similar between cachectic HCC and sham mice (Figure S2A).

**FIGURE 1:**
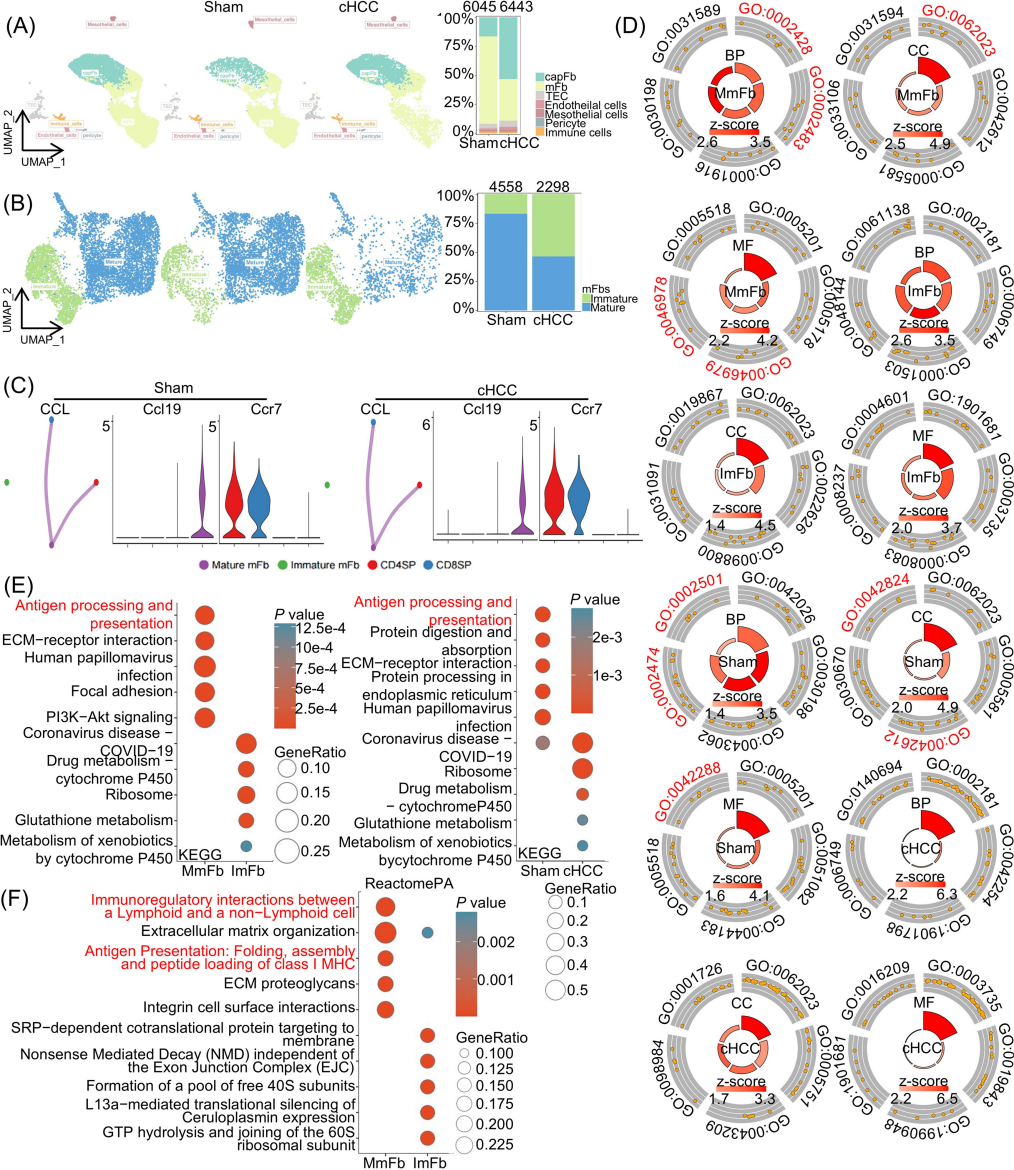
scRNA‐seq analysis revealed differences in the fibroblast composition in the thymus between sham and cachexic HCC mice. (A) A two‐dimensional representation of cells, separated by group using UMAP, and the ratio of cell types in each group displayed via a bar chart. The cells are coloured based on their type identity in the thymus of sham and cachexic HCC mice. The cell types include double‐negative thymocytes (DN), double‐positive thymocytes (DP), double‐positive blast thymocytes (DPblast), double‐positive thymocytes undergoing rearrangement (DPres), double‐positive thymocytes undergoing selection (DPsels), CD4 and CD8 single‐positive thymocytes (CD4SP and CD8SP), natural killer T cells (NKT), regulatory T cells (Treg), conventional dendritic cells (cDC), plasmacytoid dendritic cells (pDC), migratory dendritic cells (Migration DC) and macrophages and fibroblasts. (B) A two‐dimensional representation of cells, separated by group using UMAP, and the ratio of cell types in each group is shown via a bar chart. The cells are coloured based on their type identity in CD45‐thymus cells. The cell types include capsular fibroblasts (capFbs), medullary fibroblasts (mFbs), thymic epithelial cells (TECs), endothelial cells, mesothelial cells, pericytes and immune cells. (C) An interaction analysis among CD4SP, CD8SP, immature mFbs and mature mFbs in sham and cachexic HCC mice using the CellChat package (Version 1.6.1) in the CCL signalling pathway, along with the expression of pathway‐related genes among cell types. (D–F) Gene Ontology (BP), Kyoto Encyclopedia of Genes and Genomes (KEGG) (E), and ReactomePA (F) analysis of immature and mature mFbs in the thymus of sham and cachexic HCC mice. Items associated with antigen processing and presentation functions are marked in red. cHCC, cachexic hepatocellular cancer; ImFb, immature thymus medullary fibroblasts; MmFb, mature thymus medullary fibroblasts.

As thymocyte constituents were similar between cachexic HCC mice and sham mice, we focused on stromal cells. Single‐cell sequencing was performed on CD45^−^ cells in the thymi of sham (*n* = 8) and cachexic HCC (*n* = 8) mice. Consequently, after defining the cell types [12, 13, 14, 15] (Figure S3B and Table S1), a two‐dimensional projection of the total cells illustrated that fibroblasts composition changed significantly, with thymic epithelial cells (TECs), endothelial cells, mesothelial cells and pericytes unchanged (Figure 1A). Medullary fibroblasts (mFbs) decreased significantly, whereas capsular fibroblasts (capFbs expressing Dpp4, Pi16 and Mfap5) increased (Figure 1A). Mature mFbs (expressing Mmp9, Ltbp1 and Col6a5) decreased in the thymus of cachexic HCC mice (Figure 1B, Figure S3C and Table S1). Mature mFbs promote mTEC development [12]. Both mTECs and mFbs express and present self‐antigens and mediate T cell selection. Thus, the maturity disorder of mFbs in HCC mice with cachexia may lead to impaired T cell selection.

Cell communication, mainly the CCR7 (SP thymocytes)–CCL19/21 (mFbs) pathway, induces the migration of SP thymocytes to the thymic medulla, where they undergo T cell negative selection [16]. In the present study, cell communication analyses indicated that the communication strength and number of mFbs and SP thymocytes were similar in the thymus of cachexic HCC and sham mice (Figure 1C). However, communication with SP thymocytes was much stronger in mature mFbs than in immature mFbs, as indicated by both strength and number. Notably, the CCR7–CCL19/21 pathway was present only in the interaction between mature mFbs and SP thymocytes (Figures 1C and S4A). Thus, the decreased mature mFbs in HCC mice with cachexia might impair T cell negative selection.

Thymic mFbs induce negative T cell selection by presenting self‐antigens. Kyoto Encyclopedia of Genes and Genomes (KEGG) and Gene Ontology (GO) analyses revealed that mature mFbs presented increased antigen processing and presentation functions, which are also features of mFbs from the thymus of sham mice (Figure 1D,E and Tables S2–S4). ReactomePA analysis also revealed a stronger function of mature mFbs in immunoregulatory interactions between lymphoid and nonlymphoid cells and antigen presentation (Figure 1F). Thus, the maturation of mFbs might be critical for T cell negative selection. In addition, mFbs from cachexic HCC mice displayed reduced collagen genesis, as illustrated by ReactomePA (Figure S4B) and GO analyses (Figure 1D and Table S5), indicating their relative immaturity. Thus, increased immaturity of thymic mFbs may impair T cell negative selection.

### 3.2 Increased Immaturity of mFbs Is Associated With Decreased Expression of TRAs

**FIGURE 2:**
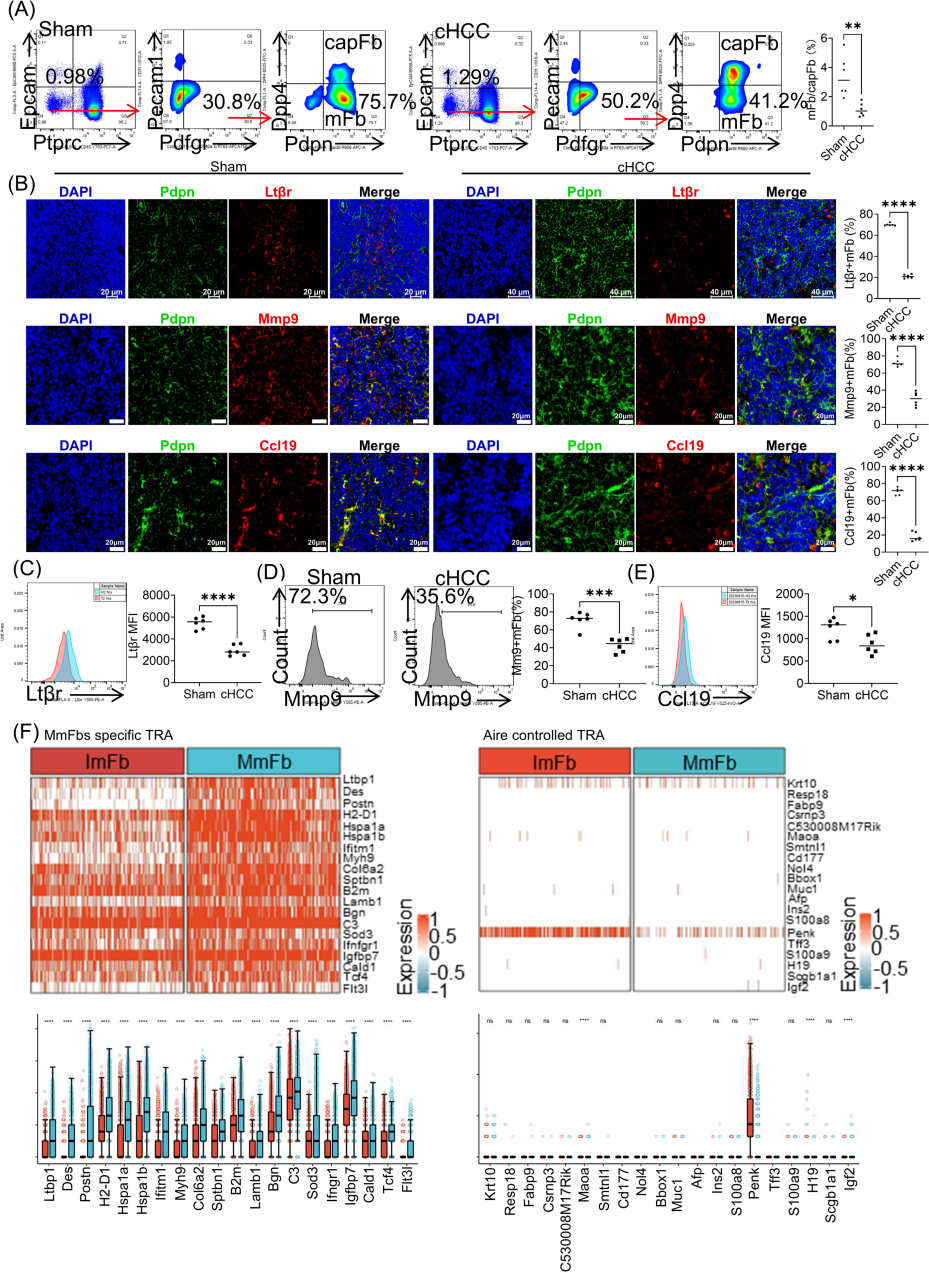
Cachexic HCC mice showed an increased ratio of immature mFbs in the thymic medulla and decreased expression of TRAs. (A) Flow cytometry analysis of the mFb/capFb ratio in cachexic HCC and sham mice. (B) Immunofluorescence analysis comparing the ratios of LtβR^+^ mFb, Mmp9^+^ mFb and Ccl19^+^ mFb in the thymic medulla of cachexic HCC and sham mice. Statistical analyses and immunofluorescence images are presented. (C, E) Flow cytometry analysis of the mean fluorescence intensity of LtβR in mFbs (C), the ratio of Mmp9^+^ mFbs (D) and the median fluorescence intensity of CCL19 (E) in mFbs in cachexic HCC and sham mice. (F) Changes in the expression of mature mFb‐specific TRA genes and Aire‐controlled TRA genes in mature and immature mFbs, presented via box plots and heatmaps. Data are presented as mean ± SD Student's *t*‐test was used to determine statistical significance. *p < 0.05, **p < 0.01, ***p < 0.001 and ****p < 0.0001.

HE staining of the total thymus revealed degradation of the thymic medulla in cachexic HCC mice (Figures S1 and S5A). Consistent with the results from single‐cell sequencing, the mFb/capFb ratio was decreased in the thymus of cachexic HCC mice (Figure 2A). Additionally, maturation of mFbs requires LtβR, which interacts with lymphotoxin (LT)‐α_1_β_2_ in SP thymocytes [12]. LtβR promotes mFb maturation by inducing the generation of multiple functional proteins, including TRAs and Mmp9 [12]. As indicated by immunofluorescence and flow cytometry, the expression of LtβR and Mmp9 decreased in mFbs in the thymus of cachexic HCC mice (Figures 2B–D and S5B), which may contribute to the immaturity of thymic mFbs.

Consistent with the results of the CCR7–CCL19/21 pathway from single‐cell sequencing, CCR7 on SP thymocytes was not altered by cachexic HCC (Figure S5C), whereas CCL19 (Figures 2B,E and S5D) on mFbs decreased. Additionally, marker genes of mature mFbs were investigated using the COSG R package, and genes with a cos_g_ score ≥ 0.6 were selected. Among these genes, *Ltbp1*, *Des*, *Postn*, *H2‐D1*, *Hspa1a*, *Hspa1b*, *Ifitm1*, *Myh9*, *Col6a2*, *Sptbn1*, *B2m*, *Lamb1*, *Bgn*, *C3*, *Sod3*, *Ifngr1*, *Igfbp7*, *Cald1*, *Tcf4* and *Flt3l* were identified as TRA‐related genes using TiGER (http://bioinfo.wilmer.jhu.edu/tiger/) (Figure 2F and Table S6). These genes were downregulated in immature mFbs, indicating impaired T cell negative selection in the thymus of cachexic HCC mice with decreased mFbs maturity. The autoimmune regulator (Aire) controls the ectopic expression of TRAs in mTECs, which is a major mechanism underlying mTEC‐induced T cell negative selection. In this study, the expression of Aire‐controlled TRAs was similar in mature and immature mFbs (Figure 2F). Above all, decreased CCL19 and TRA‐related genes of thymic mFbs might impair T cell negative selection, which is not air‐regulated.

### 3.3 Increased T‐Cell Responsiveness to Self‐Peptides in the Thymus From Cachexic HCC Mice

**FIGURE 3:**
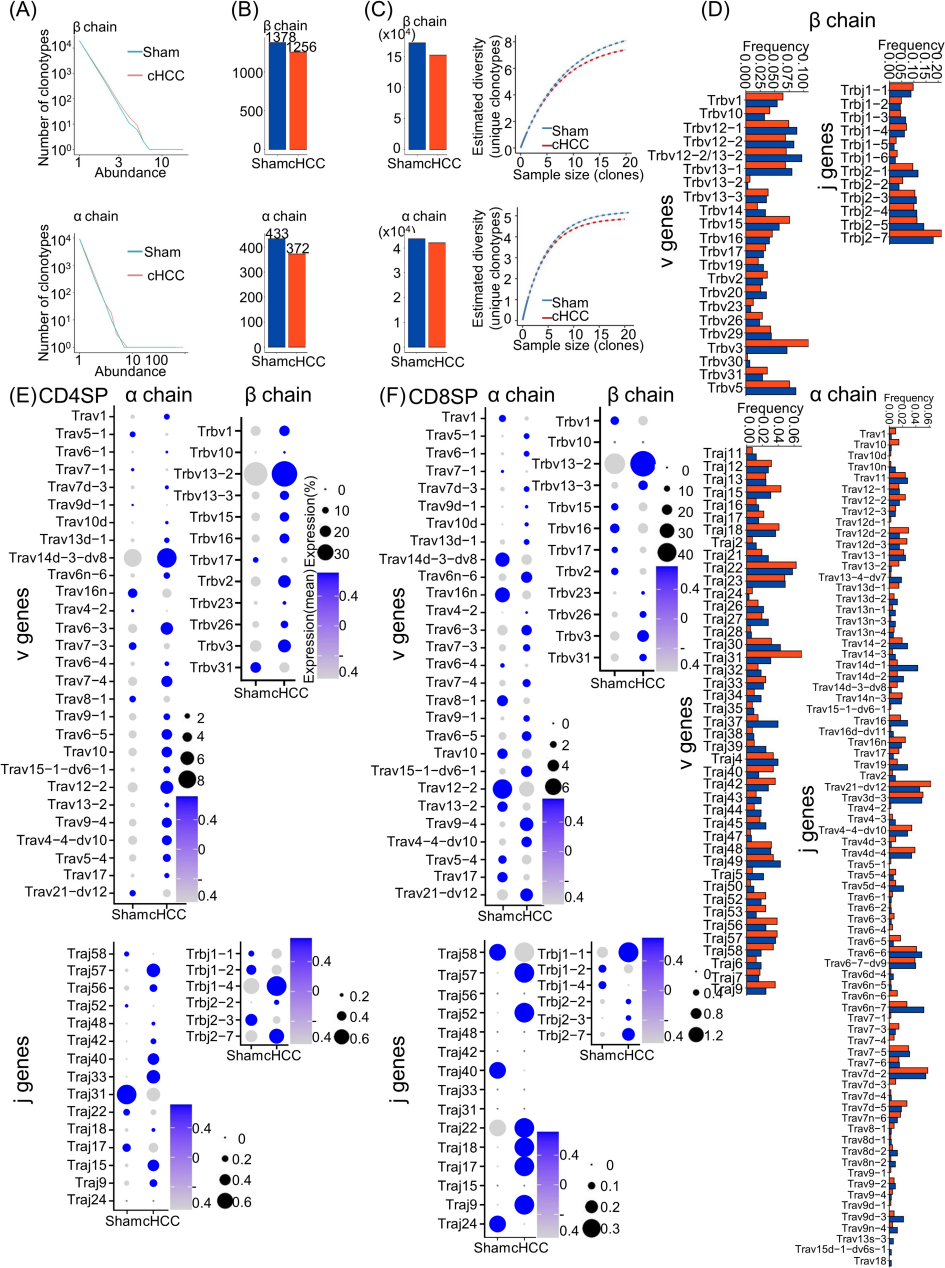
TCR analysis of thymocytes showed more frequent usage of v/j genes related to inflammatory diseases in cachexic HCC mice. (A) A line chart illustrating the number of clonotypes of v/j genes with certain abundance (clone numbers > 1) for β and α chains in the thymocytes of cachexic HCC and sham mice. (B) The number of v/j genes in the top 10% repertoires of the β and α chains in thymocytes of cachexic HCC and sham mice. (C) Diversity analysis of TCR‐β and TCR‐α repertoires in thymocytes, evaluated by the Chao1 value (left) and rarefaction analysis (right) of TCR‐β and TCR‐α repertoires in thymocytes of cachexic HCC and sham mice. (D) The frequency (usage) of expanded clonotypes of v/j genes of the β and α chains related to inflammatory diseases in thymocytes of cachexic HCC and sham mice, shown using a bar plot. (E, F) CD4 SP thymocytes (E) and CD8 SP thymocytes (F), illustrating the expression of inflammatory disease‐related v/j genes of the β and α chains in the thymocytes of cachexic HCC and sham mice in dot plots, showing the mean and percentage of expression, conducted using the Seurat package (4.4.0) in R.

LT beta receptor (LtβR)–dependent genes in mFbs include TRAs [5]. Reduced expression of LtβR in mFbs may impair the negative selection of thymocytes, potentially leading to the export of thymocytes bearing TCRs with high affinity for self‐peptides. Therefore, single‐cell TCR sequencing of thymocytes was performed. Clonotypes were defined based on their CDR3 amino acid sequences. In the β chain, there were 20 190 and 20 156 unique clonotypes in sham and cachexic HCC mice, respectively, with 9544 and 9806 unique clonotypes in the α chain (Figure S6A). The length of the CDR3 sequence in the sham and cachexic HCC mice was similar (Figure S6B), as was the frequency of usage of V/J TCR genes (Figure S6C). In the β chain, 590 (2.92%) and 701 (3.48%) unique clonotypes were amplified (clone numbers > 1) in sham and cachexic HCC mice (Figure 3A and Table S7), respectively, with 328 (3.44%) and 415 (4.23%) in the α chain (Figure 3A and Table S8). Notably, in the top 10% of repertoires, the sham group exhibited a greater variety of clonotypes than the cachexic HCC group (1378 [6.83%] vs. 1256 [6.23%] in the β chain and 433 [4.54%] vs. 372 [3.79%] in the α chain) (Figure 3B). However, the diversity index (Chao1 value and rarefaction analysis) showed a slight decrease in the diversity of the TCR‐β and TCR‐α repertoires in the cachexic HCC group (Figure 3C). Thus, certain TCR clones of the β‐ and α‐chain repertoires in the cachexic HCC group were relatively expanded. Then, their potential to induce autoimmune conditions was analysed.

A series of V/J genes have been linked to an increased susceptibility to inflammatory diseases. Therefore, the association between differentially used V/J genes in the cachexic HCC and sham groups and autoimmune diseases was investigated in the expanded clonotypes. For both the β and α chains, multiple genes related to inflammatory diseases were commonly used in the cachexic HCC group (Figure 3D). For the β chain, many V/J genes, which were highly expressed in cachexic HCC mice, were associated with type I diabetes, such as *Trbv1* [S18,19], *Trbv13‐2* [S19], *Trbj2‐7* [S19,20] and with Sjögren's syndrome, such as *Trbv3* [S21], *Trbv16* [S22] and *Trbj2‐2* [S22]. Additionally, *Trbv1* was associated with reactive arthritis [S23] and myeloperoxidase‐induced autoimmunity [S24], and *Trbv31* was linked to atherosclerosis [S25] (Figure 3D and Table S9). For the α chain, *Trav10* [S26], *Trav16n* [S20,26], *Trav17* [S27], *Trav5‐4* [S26], *Traj42* [S20,26] and *Traj53* [S26] were associated with Type I diabetes, whereas *Trav12‐2* [S28] was associated with primary biliary cholangitis. *Trav13‐2* [S29] and *Traj17* [S30] were linked to primary Sjögren's syndrome and atherosclerosis, respectively (Figure 3D and Table S9). Furthermore, many of these inflammatory disease–associated V/J genes were highly expressed (Figure 3E,F) and more frequently used (Figure S6D,E) in CD4/CD8SP thymocytes of cachexic HCC mice. These results suggest that, compared to the control group, cancer cachexia led to an increase in T cells with clonotypes associated with autoimmune diseases.

### 3.4 Cancer Cachexic CD45^+^ EPC Induced CD34^+^ Progenitor Cell Death and Contributed to Mature mFbs Disorders

**FIGURE 4:**
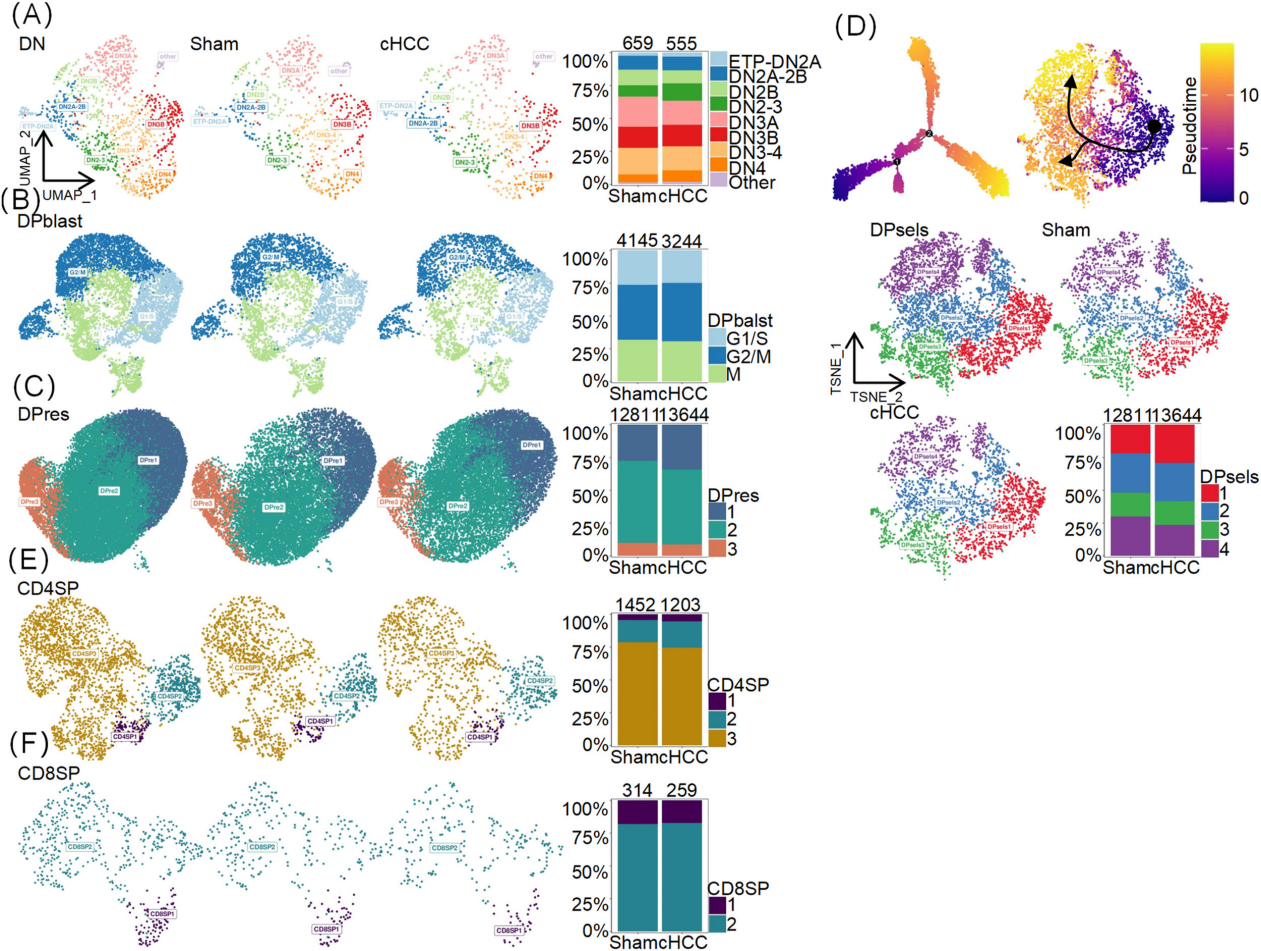
Thymocyte subtype analysis revealed more frequent usage of v/j genes associated with inflammatory diseases in cachexic HCC mice. (A) Two‐dimensional representation via UMAP/t‐SNE of cell subtypes of DN, consisting of DN1 (multipotent CD44^+^, c‐Kit^+^ and CD25^−^ early thymic progenitor cells), DN2 (stages of T lineage commitment), DN3a (γδ T cell lineage commitment), DN3b (β‐selection) and DN4 (αβ lineage–committed thymocytes that undergo cell cycle progression, initiate proliferation, and then mature into DP blasts). (B) Two‐dimensional representation via UMAP/t‐SNE of the cell subtypes of DP blasts, classified into G1/S, G2M and M subgroups according to the average expression level of all cell cycle‐associated genes. (C) Two‐dimensional representation via UMAP/t‐SNE of the cell subtypes of DPres, including DPres1, DPres2 and DPres3. (D) Two‐dimensional representation via UMAP/t‐SNE of the cell subtypes of DPsels, based on cell trajectory analysis. The trajectory of the DPsels subtypes was shown via trajectory analysis using the Monocle2 (Version 2.26.2) package. (E) Two‐dimensional representation of CD4 SP thymocyte cell subtypes via UMAP/t‐SNE, split into CD4SP1 (CD24^+^CCR7^−^), CD4SP2 (CD24^+^CCR7^+^) and CD4SP3 (CD24^−^CCR7^+^). (F) Two‐dimensional representation via UMAP/t‐SNE of the cell subtypes of CD8 SP thymocytes, classified into CD8SP1 (CD24^+^CCR7^+^) and CD8SP2 (CD24–CCR7^+^). The bar charts show the ratios of cell subtypes in each group, with cell types coloured based on their type identity.

SP thymocytes expressing LT‐α_1_β_2_ are reported to be required for mFb maturation via interaction with its receptor LtβR on mFbs [5]. Thus, maturity disorders of mFbs may be caused by thymocyte dysfunction. Therefore, the subtypes of thymocytes at different stages of development were analysed in cachexic HCC and sham mice. DN cells are the most immature stage of thymocytes, consisting of DN1 (multipotent CD44^+^, c‐Kit^+^ and CD25^−^early thymic progenitor cells), DN2 (stages of T lineage commitment), DN3a (γδ T cell lineage commitment), DN3b (β‐selection) and DN 4 (αβ lineage–committed thymocytes that undergo cell cycle progression, initiate proliferation and then mature into DP blasts) [17]. DN cell subsets were similar in the thymus of cachexic HCC and sham mice (Figures 4A and S7A). Rearrangement of the α‐chain takes place during the DP stage with strong proliferation in DP blasts, which are classified into G1/S, G2M and M subgroups according to the average expression level of all cell cycle–associated genes [S1]. The composition of DP blasts was similar in the thymi of cachectic HCC and sham mice (Figures 4B and S7B). DP blasts differentiate into DPres cells during the cell cycle and exit the cycle after division. DPres cells are categorized into three subtypes: DPres1, DPres2 and DPres3 [S1]. GO analysis revealed that DPres1 was associated with protein synthesis. DPres2, which is the subsequent state of DPres1, is involved in protein translation. DPres3 was included in the positive selection process. The composition of DPres cells was also similar in the thymi of the cachectic HCC and sham mice (Figure 4C, Figure S7C and Table S10). The DPsels represent a heterogeneous population [18]. Based on the cell trajectory analysis, DPsel cells were classified into four groups, with no significant changes observed in cachectic HCC and sham mice (Figures 4D and S7D). CD24 is a marker of the semi‐immature state of SP thymocytes, whereas CCR7, which mediates their migration, is a marker of mature SP thymocytes. CD4SP thymocytes were divided into CD4SP1 (CD24^+^CCR7^−^), CD4SP2 (CD24^+^CCR7^+^) and CD4SP3 (CD24^−^CCR7^+^) [19] and CD8SP into CD8SP1 (CD24^+^CCR7^+^) and CD8SP2 (CD24^−^CCR7^+^) (Figures 4E,F and S5E,F). The composition of SP thymocytes was also similar in the thymi of cachexic HCC and sham mice.

**FIGURE 5:**
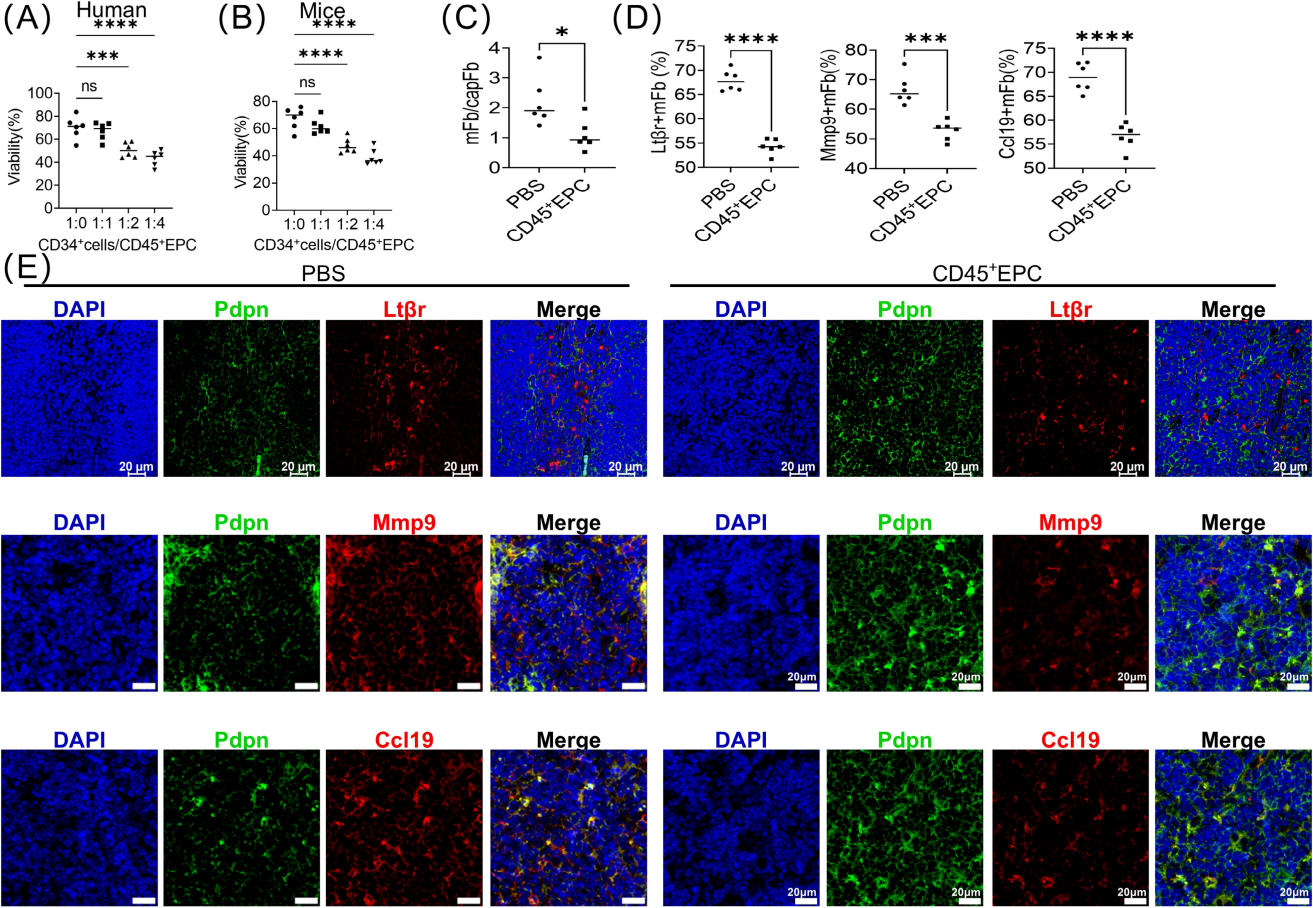
Cachexic HCC CD45^+^ EPC impairing the viability of CD34^+^ progenitors leads to mFb maturation disorder. (A, B) Effect of CD45^+^ EPCs on the viability of CD34^+^ progenitor cells via the coculture system assay, using trypan blue staining, in humans with cancer cachexia (A) and cachexic HCC mice (B). (C) Flow cytometry analysis of the mFb/capFb ratio in mice administered CD45^+^ EPCs or PBS via tail vein injection. (D, E) Immunofluorescence analysis of the ratios of LtβR^+^ mFb, Mmp9^+^ mFb and Ccl19^+^ mFb in the thymic medulla of mice administered CD45^+^ EPCs or PBS via tail vein injection. Statistical analysis (D) and immunofluorescence images (E) are shown.

H&E staining of the thymus revealed involution of the thymus (Figures S1 and S5A). Immunofluorescence analysis revealed that the proportion of mFbs (indicated by Pdpn) was higher than that of thymocytes (Figure 2B). Thus, the relative decrease in thymocytes may be due to the decrease in bone marrow–derived cell seeds in the thymus. Previous studies, including ours, have reported elevated CD45^+^ EPCs in late‐stage tumour‐bearing mice and patients with advanced cancers, suppressed T cell activation and induced endothelial cell migration [7, 9, 20]. Thus, we hypothesized that CD45^+^ EPCs might cause CD34^+^ progenitor cell dysfunction in circulation, which is reportedly the source of thymocytes [21]. The coculture system indicated that CD45^+^ EPCs from patients with HCC and cachexia caused the death of CD34^+^ progenitor cells. CD45^+^ EPCs from cachexic HCC mice displayed similar functions (Figure 5A,B). Moreover, the transfer of CD45^+^ EPCs from cachexic HCC mice intravenously to tumour‐free mice caused a decrease in thymocytes compared to mFbs, decreased the mFb/capFb ratio (Figure 5C) and decreased Mmp9, LtβR and Ccl19 expression in mFbs (Figure 5D,E). Thus, cancer cachexic CD45^+^ EPC induced CD34^+^ progenitor cell death and consequently decreased thymocytes, which finally contributed to the maturation disorder of mFbs.

### 3.5 Cachexic HCC Mice Display Impaired CD24^+^CD4^+^CD8^−^SP Thymocyte Negative Selection and an Autoimmune Response

**FIGURE 6:**
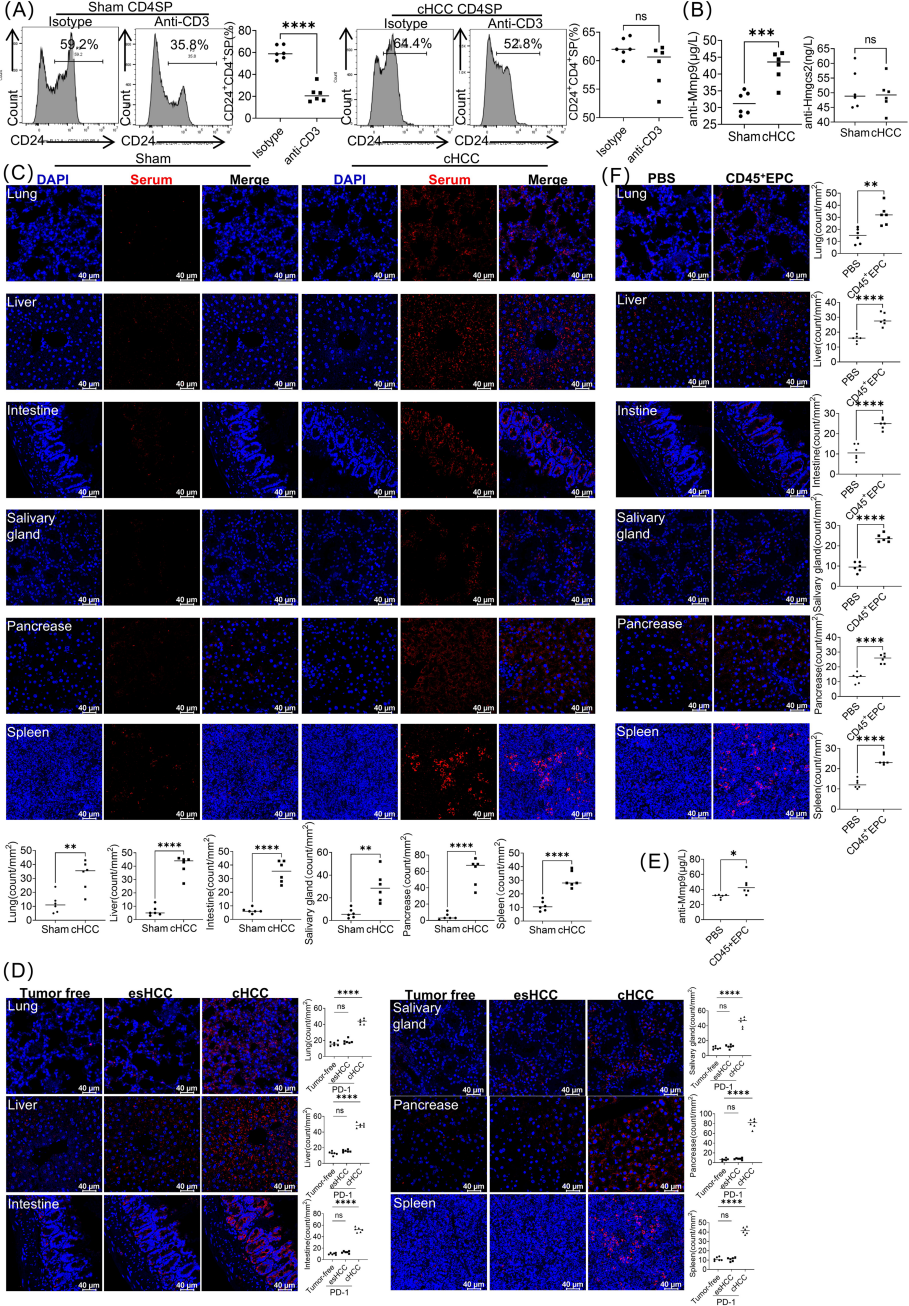
Cachexic HCC mice exhibit dysfunction in T cell negative selection in the thymus. (A) Flow cytometric analysis of the ratio of CD24^+^CD4^+^CD8–SP thymocytes in cachexic HCC and sham mice following intraperitoneal injection of anti‐CD3 mAb (10 mg/kg, clone 17A2, BioLegend) or isotype controls. (B) The amount of serum Mmp9 and Hmgcs2 autoantibodies in cachexic HCC and sham mice was measured via ELISA. (C) Immunofluorescence analysis of autoantibodies in multiple organ sections of Rag1^−/−^ mice incubated with serum from sham or cachexic HCC mice. Statistical analysis (bottom) and representative immunofluorescence images (top) are shown. (D) Immunofluorescence analysis of autoantibodies in multiple organ sections from Rag1^−/−^ mice incubated with serum from tumour‐free mice, mice with early‐stage HCC (esHCC), and cachexic HCC (cHCC) mice administered anti‐PD‐1 (10–12.5 mg/kg, clone RMP1‐14, Bio X Cell) via intraperitoneal injection. Representative immunofluorescence images and statistical analyses are shown. (E) ELISA of Mmp9 autoantibody levels in tumour‐free mice administered CD45^+^ EPCs from cachexic HCC mice or PBS via tail vein injection. (F) Immunofluorescence analysis of autoantibodies in multiple organ sections from Rag1^−/−^ mice incubated with serum from tumour‐free mice, which were administered CD45^+^ EPCs from cachexic HCC mice or PBS via tail vein injection. Representative immunofluorescence images and statistical analyses are shown.

The CCR7/CCL19 pathway mediates the negative selection of CD24^+^CD4^+^CD8^−^SP thymocytes [16]. Intravenous administration of anti‐CD3 mAb induced negative selection of T cells in the thymus. In this study, anti‐CD3 mAb reduced the number of CD24^+^CD4^+^CD8^−^SP thymocytes in the sham mice. However, the number of CD24^+^CD4^+^CD8^−^SP thymocytes did not decrease significantly in the thymus of the cachexic HCC mice (Figure 6A). Thus, the negative selection of CD24^+^CD4^+^CD8^−^SP thymocytes in cachexic HCC mice was impaired.

Mmp9 and Hmgcs2 are representative TRAs predominantly expressed in mFbs in an LtβR‐dependent manner [12]. Ltbr^−/−^ mice contain higher levels of Mmp9 and Hmgcs2 autoantibodies [12]. In this study, the level of Mmp9 autoantibodies in the serum of cachexic HCC mice was higher than that in the serum of sham mice, whereas the level of Hmgcs2 autoantibodies remained unchanged (Figure 6B). In addition, cachexic HCC mice displayed a marked production of autoantibodies against the lung, liver, intestine, salivary gland, pancreas and spleen compared to sham mice (Figures 6C and S8A). Administration of an anti‐PD‐1 antibody to cachectic HCC mice accelerated the production of autoantibodies targeting multiple organs, compared with mice in the early stages of HCC (Figures 6D and S8B). Consequently, autoantibody levels were elevated in cachectic HCC mice.

Moreover, intravenous transfer of CD45^+^ EPCs from cachexic HCC mice to tumour‐free mice caused elevation of Mmp9 autoantibodies in the serum (Figure 6E) and marked production of autoantibodies against the lung, liver, intestine, salivary gland, pancreas and spleen (Figures 6F and S8C). Cancer cachexic CD45^+^ EPC promote the generation of autoimmune antibodies.

### 3.6 Serum Autoantibodies Against Tumour Predicted Antitumour Efficacy of ICIs

**FIGURE 7:**
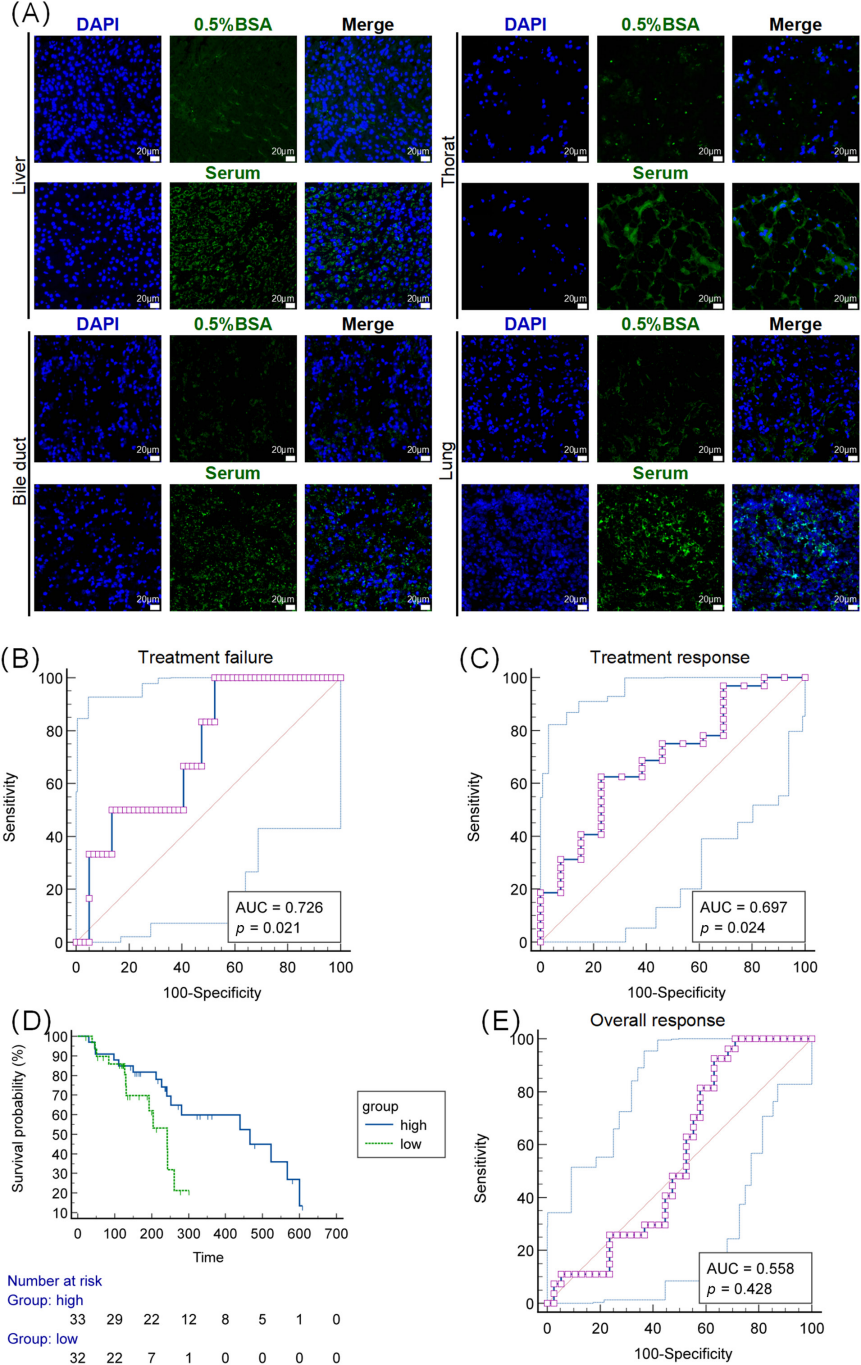
Serum autoantibodies against tumours predict the antitumour efficacy of ICIs among patients with cancer. (A) Immunofluorescence analysis of autoantibodies against tumour tissues in the serum of patients with cancer cachexia. (B, C) ROC analysis of the amount of serum autoantibodies against tumour tissues to predict treatment failure (progressive disease) (B) and a duration of treatment response > 180 days (C). (D) PFS of patients with low or high levels of serum autoantibodies. (E) ROC analysis of the amount of serum autoantibodies against tumour tissues to predict the overall response.

The clinical significance of the serum autoantibodies was then analysed. Sixty‐five patients with advanced or locally advanced cancer who received ICI treatment were included in this study (Table S11). To explore the production of autoantibodies, tumour tissue sections were incubated with sera from patients receiving two cycles of ICI treatment. A certain number of autoantibodies against TRAs in patient tumour tissues were found in their serum (Figure 7A). Seven patients developed irAEs. Univariate Cox regression analysis showed that clinical parameters, including cachexia (BMI < 20 kg/m^2^ or weight loss ≥ 5% over the past 6 months), failed to predict treatment failure. However, patients with low levels of autoantibodies had a higher risk of disease progression (HR, 2.39; 95% CI [1.02–5.63], *p* = 0.046) (Table S12). The number of serum autoantibodies against tumour tissues was used to predict treatment efficacy (Table S12). ROC curves indicated that the amount of serum autoantibodies against tumour tissues predicted treatment failure (Figure 7B) and long‐term (> 180 days) treatment response (Figure 7C). Patients with high levels of serum autoantibodies against tumours showed a favourable PFS (Figure 7D). However, the number of serum autoantibodies against the tumour did not predict the overall response (Figure 7E).

## 4 Discussion

Cancer cachexia affects approximately 70% of patients with cancer and is characterized by weight loss and skeletal muscle mass with or without loss of fat mass [22]. Cancer cachexia leads to reduced physical function, resistance to anticancer therapy and decreased survival [23]. However, in the era of immunotherapy, the effects of cancer cachexia on the efficacy and adverse effects of ICIs remain unclear. Pro‐inflammatory factors, MDSCs and activated T cells participate in the development of cancer cachexia [24]. However, the effects of cancer cachexia on the thymus remain unclear. Thymus involution occurs in adult humans [25]. Thymic involution, especially age‐related involution, is characterized by disruption of tissue architecture, reduction in thymic mass, thymocyte numbers, generation of naive T cells reaching the periphery and diversity of the peripheral T cell repertoire [26]. In addition, several factors have been reported to induce thymic involution, including chemotherapy, ionizing radiation exposure and severe infections [2]. Owing to the discrepancy in the mechanism inducing thymus involution, thymus dysfunction varies under different pathological conditions. This study indicated that cancer cachexia caused maturity disorders in thymic mesenchymal fibroblasts, restricting the presentation of TRA, which caused impaired T cell negative selection. The TCR repertoire against TRA increased, which mediated the adverse and favourable effects of ICIs as anticancer treatments.

Previous studies have found thymus atrophy in older mice, accompanied by thymic architectural changes [27]. A decline in the expression of TEC‐specific markers and an increase in fibroblast content in the aging mouse thymus have been reported [27]. However, the maturation of thymic fibroblasts in older mice has not been investigated. The thymus of older mice exhibited increased senescence and apoptosis [27], indicating significant changes in thymocytes, which influenced the development of thymic fibroblasts. A previous study also found infiltration of inflammatory cells into multiple organs of older mice. However, cancer cachexia–induced thymus atrophy may represent a distinct condition [25]. Thus, aging‐induced thymus atrophy may play a different role in the efficacy and adverse effects of ICI treatment.

Cancer‐associated CD45^+^ EPCs' elimination from thymocyte cell seeds may contribute to a reduction in thymocytes, which in turn inhibits mFb maturation. Fibroblasts are activated in remote tumour‐free organs by extracellular vesicles and form a pre‐metastatic niche for metastasis [28]. In this study, mFb in the thymus of mice with cancer cachexia presented a maturity disorder instead of pathological activation. SP thymocytes contribute to mFb maturation through interactions with LtβR and LTs [5]. In the present study, we observed a relative decrease in the number of thymocytes in the cancer cachexia group. Thus, we speculated that thymocyte dysfunction may be the cause of mFb maturity disorders. However, cell development in all subpopulations of DN, DP and SP thymocytes in the thymus of cancer cachexia and tumour‐free mice was similar. Thus, we speculated that the incorporation of cell seeds into the thymus may be a linchpin. Anaemia is a common complication of cancer cachexia and is a sign of maintenance [29, 30]. Extramedullary haematopoiesis occurs during cancer and anaemia [7, 31, 32] and is reportedly ineffective, leading to the accumulation of CD45^+^ EPCs in circulation [33, 34]. Cancer‐associated CD45^+^ EPCs suppress T cell activation in circulation and the tumour microenvironment and induce vascular endothelial cell (VEC) migration [9]. However, their presence in the thymus of cachexic HCC mice was not observed in the present study. Moreover, we found that cancer‐associated CD45^+^ EPCs induced the death of CD34^+^ cells, which are considered thymocyte cell seeds. Decreased supplementation of source cells may be a critical mechanism underlying the maturity disorder of mFbs. However, further studies are required to investigate the causes of thymic involution in cancer cachexia.

Cancer cachexia is an unfavourable prognostic factor for survival and has a substantial negative impact on the response to ICI therapy [35, 36]. However, a significant proportion of patients with cancer cachexia respond well to ICIs and exhibit reversible cachexia [37]. In addition, the association between the adverse effects of ICIs and cancer cachexia remains unclear. Above all, cancer cachexia may be a double‐edged sword for the treatment of ICI. In this study, serum autoantibodies accumulated in mice with cancer cachexia and were accelerated after anti‐PD‐1 antibody treatment. The TCR repertoire against TRA was increased in the thymocytes of mice with cancer cachexia. These results indicated that cancer cachexia may promote irAEs to some extent. Serum autoantibodies against tumour tissues predicted the efficacy of combined ICI treatment. Patients with low levels of serum autoantibodies against tumour tissues showed increased treatment failure. Thus, maturity disorders of mFb due to cancer cachexia and the consequence of T cell negative selection failure might mediate the effects of ICIs.

This study found that impaired cell negative selection led to the accumulation of autoantibodies in the serum, indicating antigen‐specific crosstalk between T and B cells. Previous studies have found antigen‐specific T follicular helper cells, which might be involved in the mechanism [38, 39]. Further studies are required to elucidate the underlying mechanisms. However, the mechanisms and clinical applications of these phenomena require further investigation. Although this study found cancer‐associated CD45^+^ EPC‐mediated maturity disorders of mFbs in the thymus, the number of CD45^+^ EPCs is still an unfavourable prognostic factor in patients with cancer [40, 41]. Therefore, the clinical applications of CD45^+^ EPCs require further investigation. In this study, we investigated serum antibodies reactive to antigens within the tumour, rather than autoimmune antigens within normal tissues. Thus, it is not feasible to investigate the association between serum autoantibody levels and irAEs. Additionally, the generation of antitumour antibodies, particularly against TSAs, is not due to cancer cachexia–induced thymus atrophy. The present study found that serum antibody levels have predictive value for treatment failure of ICIs, highlighting the clinical potential of serum antibodies. However, this result did not confirm the hypothesis that cancer cachexia increases the production of autoantibodies. Further studies using novel methodologies to investigate serum autoantibodies are necessary to confirm these hypotheses. Moreover, cancer cachexia not only affects the thymus but also influences T and B cells, complicating its effect on the efficacy and adverse effects of ICIs. Future studies aimed at predicting the efficacy and adverse effects of ICIs should incorporate cachectic parameters.

## 5 Conclusion

This study found that cancer cachexia caused maturity disorders of the thymic mFbs, restricted the presentation of TRA and impaired T cell negative selection. The TCR repertoire against TRA increased, and it was associated with adverse and favourable effects of ICIs. The cancer‐associated elimination of CD45^+^ EPCs from thymocyte seeds may contribute to the reduction of thymic involution.

## Ethics Statement

This study was approved by the Ethics Committee of the Third Affiliated Hospital of Sun Yat‐sen University (Permit Number [2020]02‐452).

## Consent

All participants provided written informed consent prior to sampling in accordance with the principles of the Declaration of Helsinki. All animal experiments complied with the ARRIVE guidelines and were carried out in accordance with the U.K. Animals (Scientific Procedures) Act, 1986, and associated guidelines; the EU Directive 2010/63/EU for animal experiments; and the National Research Council's Guide for the Care and Use of Laboratory Animals. Animal experiments were approved by the Institutional Animal Care and Use Committee of the Third Affiliated Hospital of Sun Yat‐sen University.

## Conflicts of Interest

The authors declare no conflicts of interest.

## Supporting information


**Figure S1** Body weights, tumour burden, thymus weight and cell number of sham (*n* = 8) and cachexic HCC (*n* = 8) mice.

**Figure S2.** Identification of cell types. (A) Two‐dimensional representation of cells and cells split by group via umap and ratio of cell types in each group via bar chart, which were coloured based on cell type identity in the whole thymocytes. (B) marker genes of DN, DPblast, DPres, DPsels, CD4SP, CD8SP, NKT, Treg, γδT, B cells, cDC, pDC, migration DC, macrophages and fibroblasts projected onto umap plots.

**Figure S3.** Identification of cell types (A) Dot plot displaying marker genes of DN, DPblast, DPres, DPsels, CD4SP, CD8SP, NKT, Treg, γδT, B cells, cDC, pDC, migration DC, macrophages and fibroblasts. (B, C) Marker genes of capFb, mFb, TEC, endothelial cells, mesothelial cells, pericytes, immune cells (B), immature mFbs and mature mFbs (C) projected onto umap plots.

**Figure S4**. (A) Interaction analysis among CD4SP, CD8SP, immature mFbs and mature mFbs in sham and cachexic HCC mice by the cellChat package (Version 1.6.1) in R. Number and weight of cell–cell interactions. (B) ReactomePA analysis of mFbs of the thymus in sham mice and cachexic HCC mice. Items associated with antigen processing and presentation functions are marked in red.

**Figure S5.** Supplementary results in Figure 2. (A) Representative haematoxylin–eosin (H&E) staining images showing changes in the thymic medulla in cachexic HCC and sham mice. White arrows indicate the corticomedullary junction. (B) Sequential gating strategy for Mmp9^+^ mFb identification. (C) Flow cytometry analysis of the expression of CCR7 on CD4/8 SP thymocytes in sham and cachexic HCC mice. (D) Sequential gating strategy for Ccl19^+^ mFb identification.

**Figure S6.** Usage of v/j genes of thymocytes. (A) The number of unique clonotypes of TCR‐β and TCR‐α repertoires in thymocytes of cachexic HCC and sham mice. (B) CDR3 length distribution of the β and α chains in the thymocytes of cachexic HCC and sham mice. (C) Comparison of the usage of the v/j gene of the β and α chains in the thymocytes of cachexic HCC and sham mice. (D) Comparison of the usage of v/j genes of the β and α chains in CD4SP thymocytes of cachexic HCC and sham mice. (E) Comparison of the usage of the v/j gene of the β and α chains in CD8SP thymocytes of cachexic HCC and sham mice. The analysis was performed by using the Immunarch package (1.0.0) in R.

**Figure S7.** Thymocyte subtypes analysis. (A) Two‐dimensional representation (via umap) of the score of marker genes associated with the differential stage of DN thymocytes using the AUC package (Version 1.20.2). Heatmaps show correlation (via psych package, Version 2.3.9) of gene expression profiles between bulk‐sorted thymocyte subpopulations (GSE15907) and our single‐cell clusters (resolution.1.7, left) and annotated thymocyte types (right), respectively. (B) Two‐dimensional representation (via umap) of the score of marker genes of cell cycles using the AUC package (1.20.2). Comparisons of cell cycle score using the AUC package (1.20.2) in different single‐cell clusters (resolution.0.7, left) and different annotated DPblasts subtypes (right), respectively. (C) Gene ontology analysis of the top 50 marker genes from each DPres thymocyte subtype using FindAllMarkers functions in the Seurat (4.4.0) and clusterProfiler (4.6.2) packages. (D) Distribution of each cluster (resolution.1.0) of DPsels thymocytes in trajectory via trajectory analysis by monocle (2.26.2) package. Cluster 9 was deleted for belonging to contaminated cells. (E, F) Two‐dimensional representation via umap of clusters and expression of CD24 and CCR7 in CD4SP (up) and CD8SP (down) thymocytes.

**Figure S8.** Immunofluorescence supplementation results in Figure 6. (A) Immunofluorescence analysis of autoantibodies combination on kidney and fat sections and statistical results in Rag1^−/−^ mice incubated with serum from sham or cachexic HCC mice. (B) Representative immunofluorescence images of multiple organ sections from Rag1^−/−^ mice in Figure 5D. Statistical results of kidney and fact sections are shown. (C) Representative immunofluorescence images of multiple organ sections from Rag1^−/−^ mice in Figure 5E.

**Table S1.** Marker genes of various cell types.
**Table S2.** Gene ontology analysis of the top 100 marker genes of mature mFbs.
**Table S3.** Gene ontology analysis of the top 100 marker genes of immature mFbs.
**Table S4.** Gene ontology analysis of the top 100 marker genes of mFbs from sham mice.
**Table S5.** Gene ontology analysis of the top 100 marker genes of mFbs from cachexic HCC mice.
**Table S6.** Tissue‐specific expression of marker genes (COSG score ≥ 0.6) of mature mFbs.
**Table S7.** Distribution of colontype abundances in TCR‐β repertoire.
**Table S8.** Distribution of colontype abundances in TCR‐α repertoire.
**Table S9.** Inflammatory diseases associated with V/J genes.
**Table S10.** Gene Ontology analysis of DPres subtypes.
**Table S11.** Clinical characteristics of patients with advanced or locally advanced cancer treated with PD‐1/L1 antibody.
**Table S12.** Factors affecting disease progression by univariate Cox regression analysis.
**Table S13.** REAGENT or RESOURC.

## Data Availability

Data will be made available upon request.
