# Supplementary figures and images for: Thymic Microenvironment Remodeling in Cancer Cachexia as a Determinant of Checkpoint Inhibitor Efficacy and Toxicity
Source: J Cachexia Sarcopenia Muscle. 2025 Jul 16;16(4):e13874. doi: 10.1002/jcsm.13874 (PMC12264390; doi:10.1002/jcsm.13874)

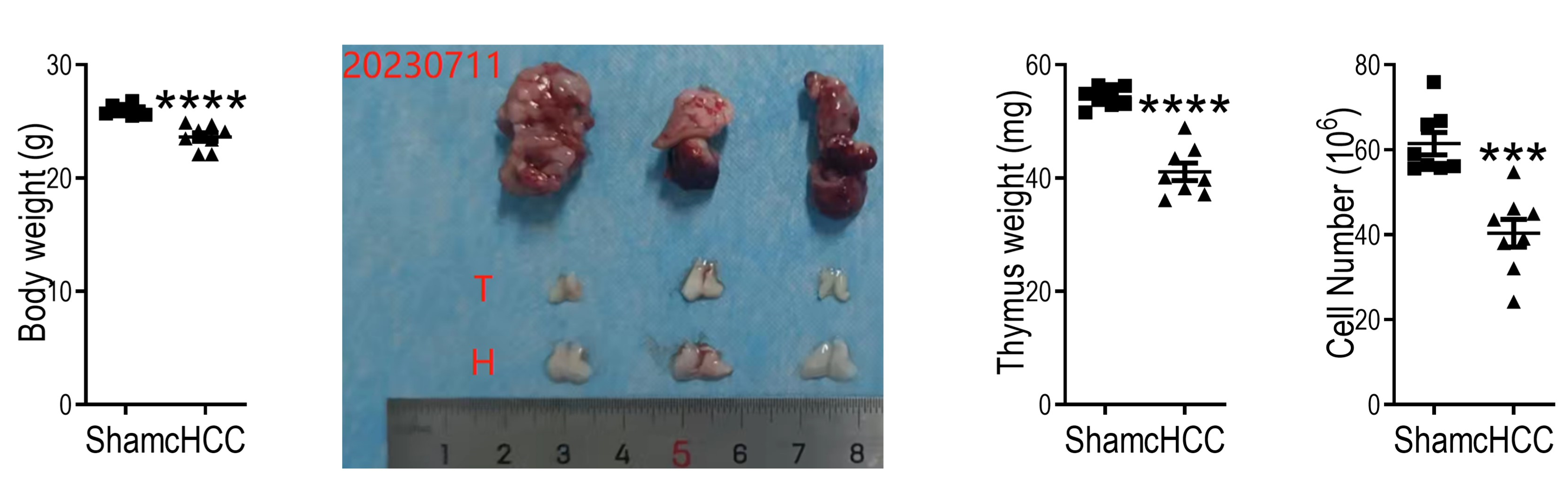

Supplement: Supplementary file 1 — Figure S1 Body weights, tumour burden, thymus weight and cell number of sham (n = 8) and cachexic HCC (n = 8) mice. [file JCSM-16-e13874-s004.tif]

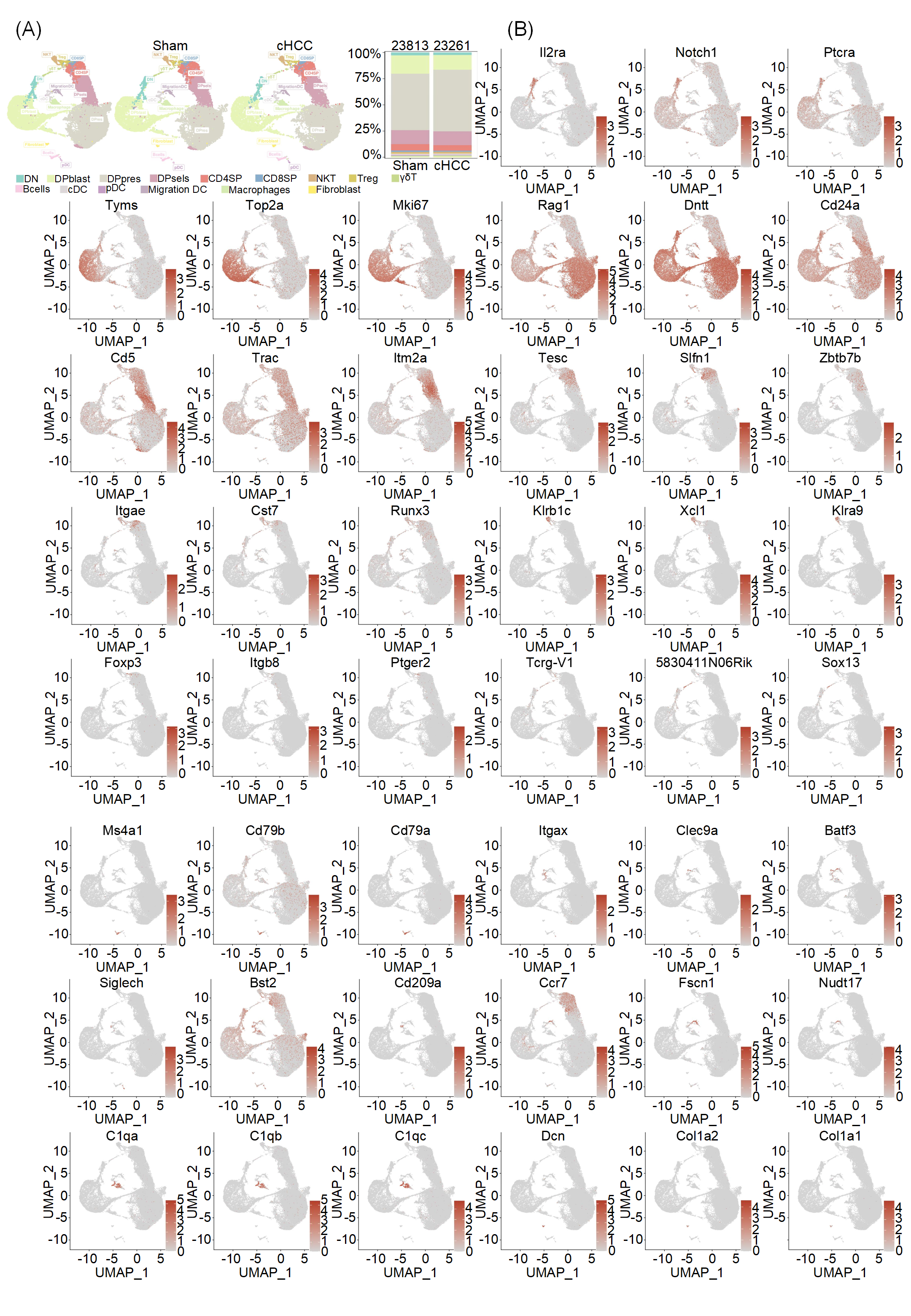

Supplement: Supplementary file 2 — Figure S2. Identification of cell types. (A) Two‐dimensional representation of cells and cells split by group via umap and ratio of cell types in each group via bar chart, which were coloured based on cell type identity in the whole thymocytes. (B) marker genes of DN, DPblast, DPres, DPsels, CD4SP, CD8SP, NKT, Treg, γδT, B cells, cDC, pDC, migration DC, macrophages and fibroblasts projected onto umap plots. [file JCSM-16-e13874-s008.tif]

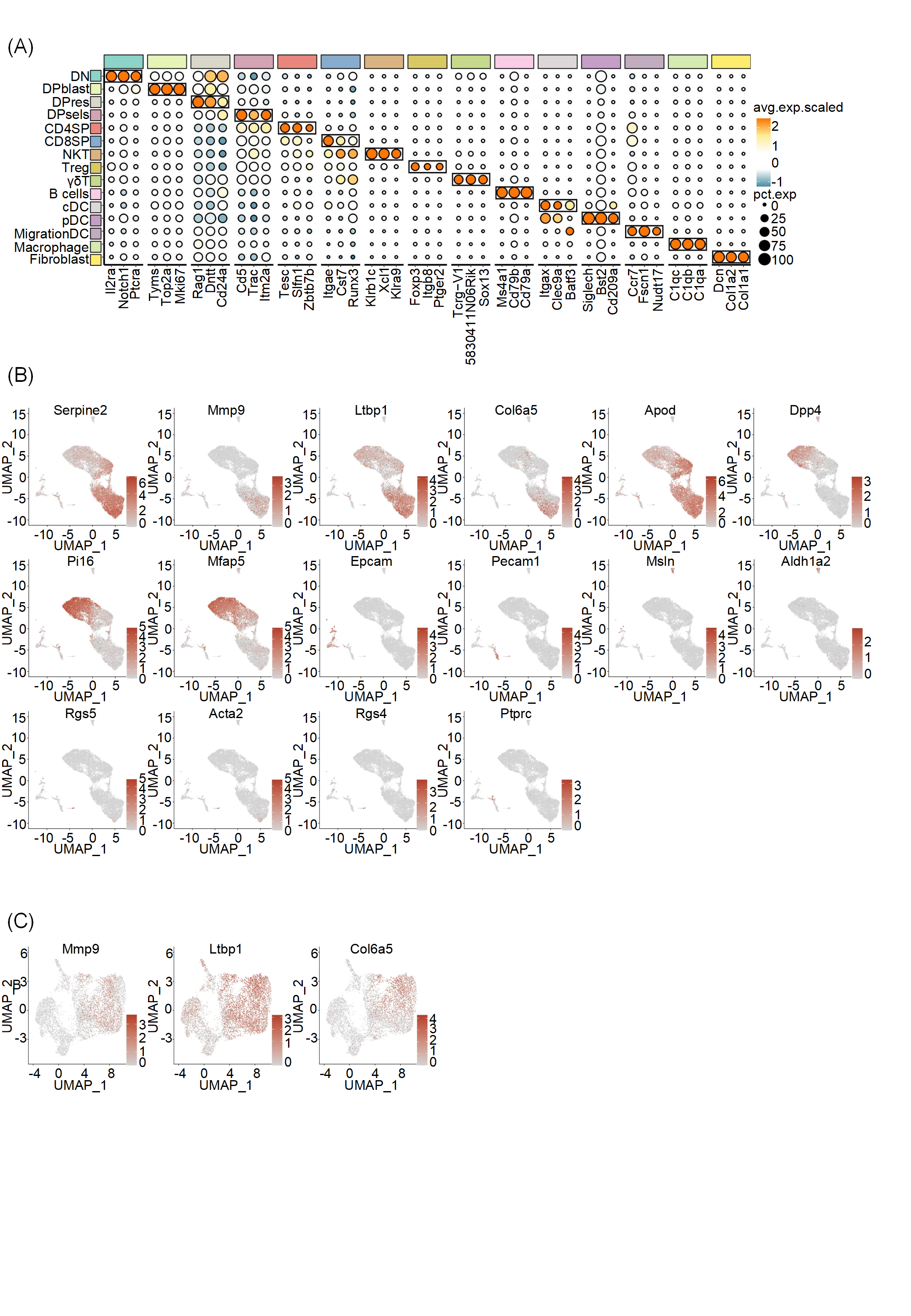

Supplement: Supplementary file 3 — Figure S3. Identification of cell types (A) Dot plot displaying marker genes of DN, DPblast, DPres, DPsels, CD4SP, CD8SP, NKT, Treg, γδT, B cells, cDC, pDC, migration DC, macrophages and fibroblasts. (B, C) Marker genes of capFb, mFb, TEC, endothelial cells, mesothelial cells, pericytes, immune cells (B), immature mFbs and mature mFbs (C) projected onto umap plots. [file JCSM-16-e13874-s001.tif]

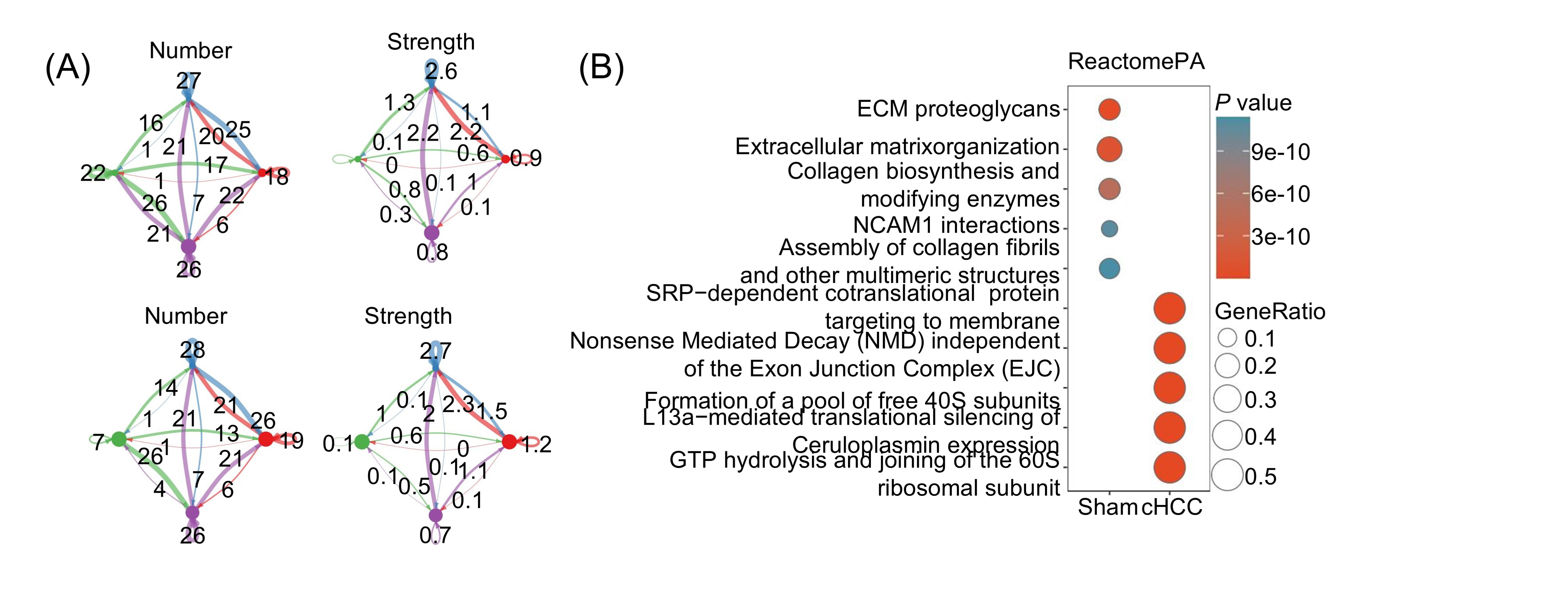

Supplement: Supplementary file 4 — Figure S4. (A) Interaction analysis among CD4SP, CD8SP, immature mFbs and mature mFbs in sham and cachexic HCC mice by the cellChat package (Version 1.6.1) in R. Number and weight of cell–cell interactions. (B) ReactomePA analysis of mFbs of the thymus in sham mice and cachexic HCC mice. Items associated with antigen processing and presentation functions are marked in red. [file JCSM-16-e13874-s002.tif]

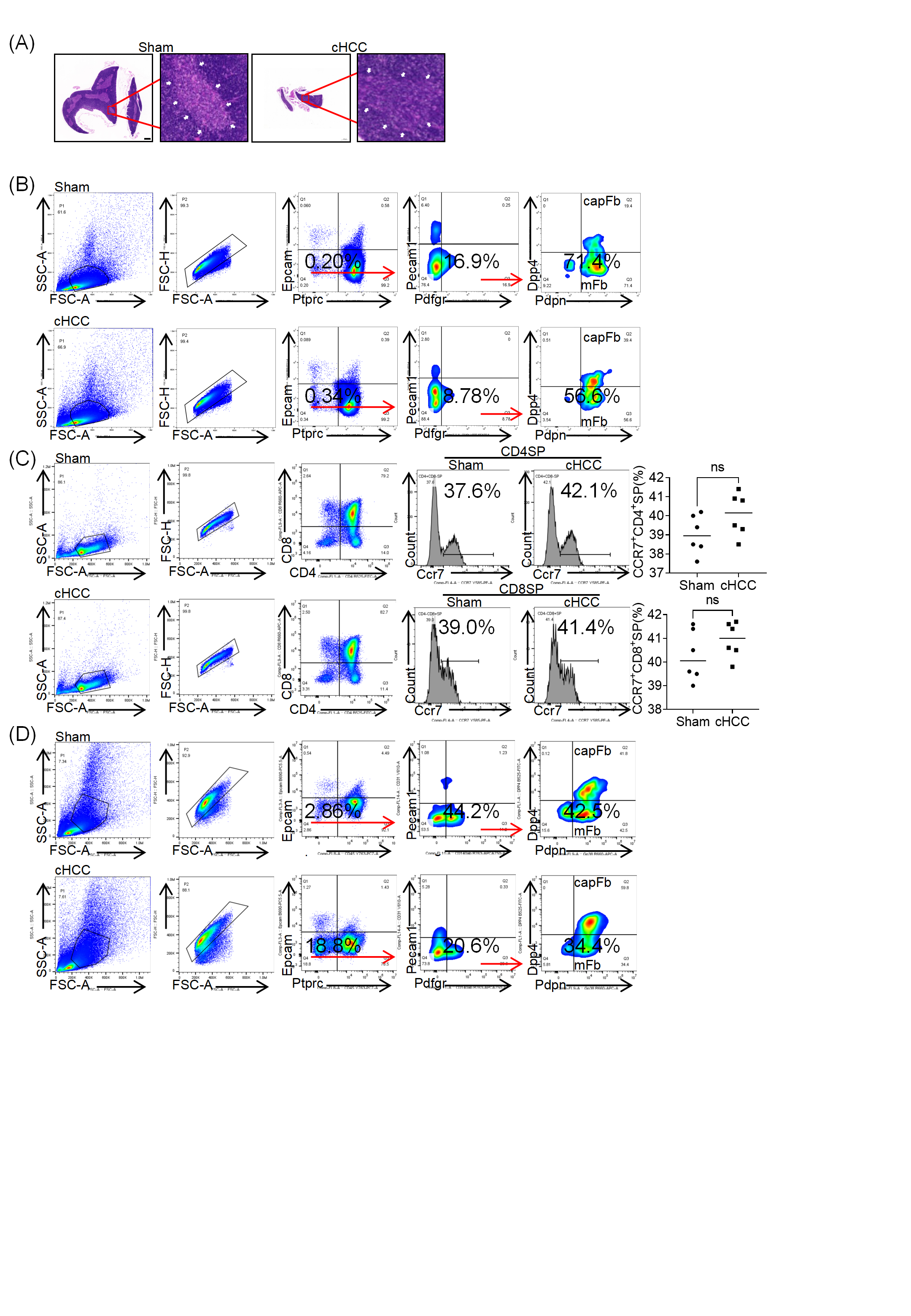

Supplement: Supplementary file 5 — Figure S5. Supplementary results in Figure 2. (A) Representative haematoxylin–eosin (H&E) staining images showing changes in the thymic medulla in cachexic HCC and sham mice. White arrows indicate the corticomedullary junction. (B) Sequential gating strategy for Mmp9+ mFb identification. (C) Flow cytometry analysis of the expression of CCR7 on CD4/8 SP thymocytes in sham and cachexic HCC mice. (D) Sequential gating strategy for Ccl19+ mFb identification. [file JCSM-16-e13874-s007.tif]

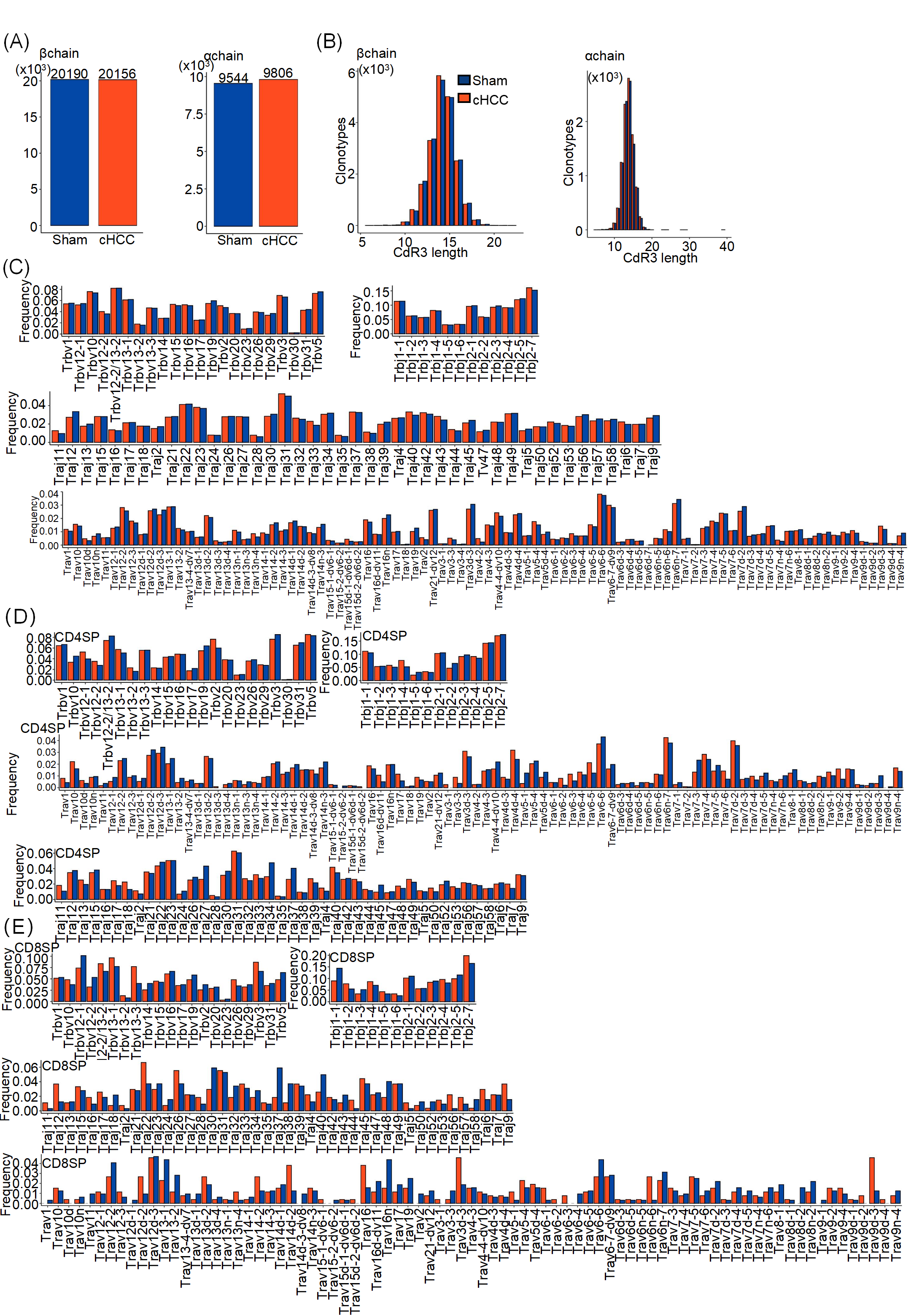

Supplement: Supplementary file 6 — Figure S6. Usage of v/j genes of thymocytes. (A) The number of unique clonotypes of TCR‐β and TCR‐α repertoires in thymocytes of cachexic HCC and sham mice. (B) CDR3 length distribution of the β and α chains in the thymocytes of cachexic HCC and sham mice. (C) Comparison of the usage of the v/j gene of the β and α chains in the thymocytes of cachexic HCC and sham mice. (D) Comparison of the usage of v/j genes of the β and α chains in CD4SP thymocytes of cachexic HCC and sham mice. (E) Comparison of the usage of the v/j gene of the β and α chains in CD8SP thymocytes of cachexic HCC and sham mice. The analysis was performed by using the Immunarch package (1.0.0) in R. [file JCSM-16-e13874-s003.tif]

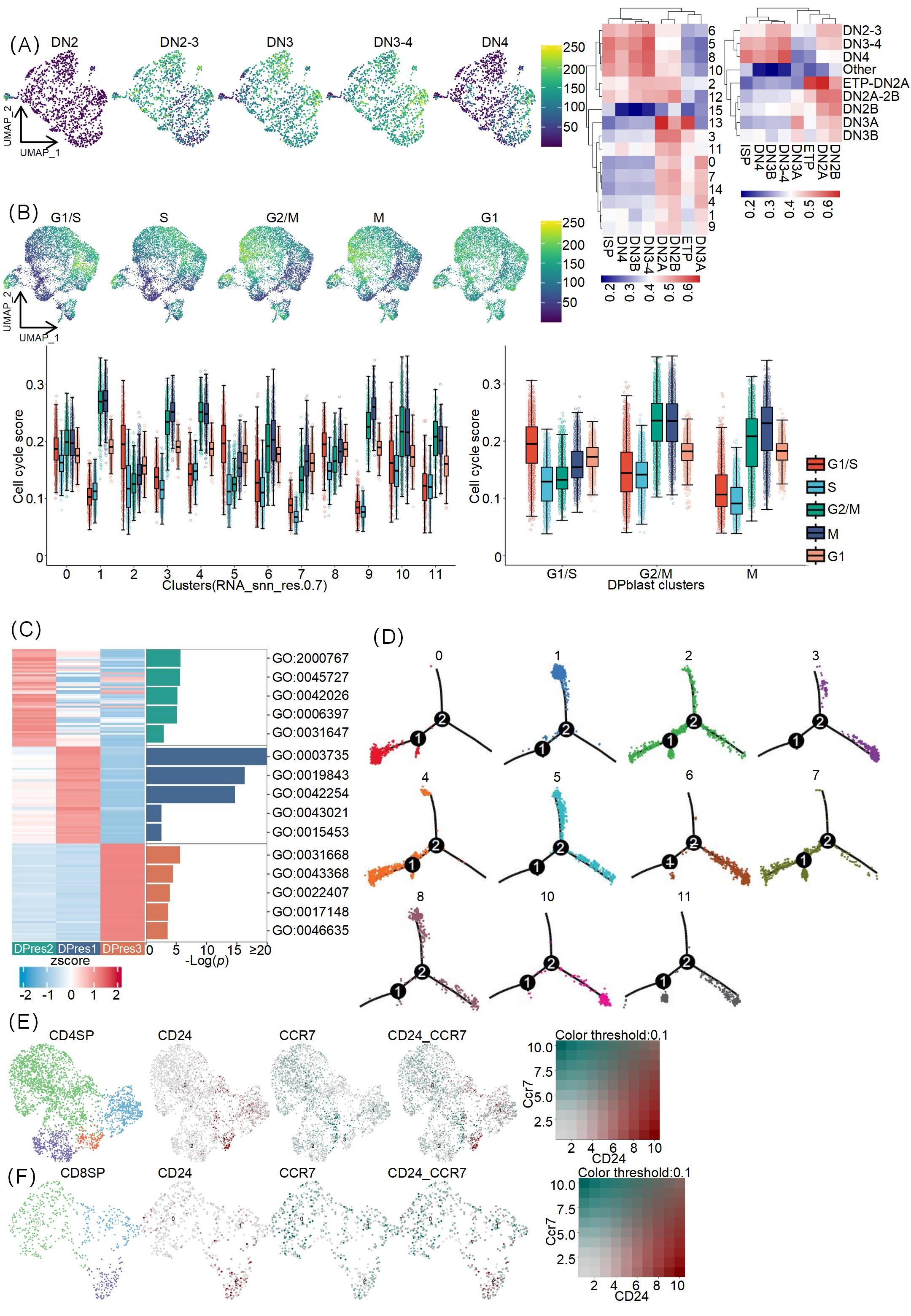

Supplement: Supplementary file 7 — Figure S7. Thymocyte subtypes analysis. (A) Two‐dimensional representation (via umap) of the score of marker genes associated with the differential stage of DN thymocytes using the AUC package (Version 1.20.2). Heatmaps show correlation (via psych package, Version 2.3.9) of gene expression profiles between bulk‐sorted thymocyte subpopulations (GSE15907) and our single‐cell clusters (resolution.1.7, left) and annotated thymocyte types (right), respectively. (B) Two‐dimensional representation (via umap) of the score of marker genes of cell cycles using the AUC package (1.20.2). Comparisons of cell cycle score using the AUC package (1.20.2) in different single‐cell clusters (resolution.0.7, left) and different annotated DPblasts subtypes (right), respectively. (C) Gene ontology analysis of the top 50 marker genes from each DPres thymocyte subtype using FindAllMarkers functions in the Seurat (4.4.0) and clusterProfiler (4.6.2) packages. (D) Distribution of each cluster (resolution.1.0) of DPsels thymocytes in trajectory via trajectory analysis by monocle (2.26.2) package. Cluster 9 was deleted for belonging to contaminated cells. (E, F) Two‐dimensional representation via umap of clusters and expression of CD24 and CCR7 in CD4SP (up) and CD8SP (down) thymocytes. [file JCSM-16-e13874-s005.tif]

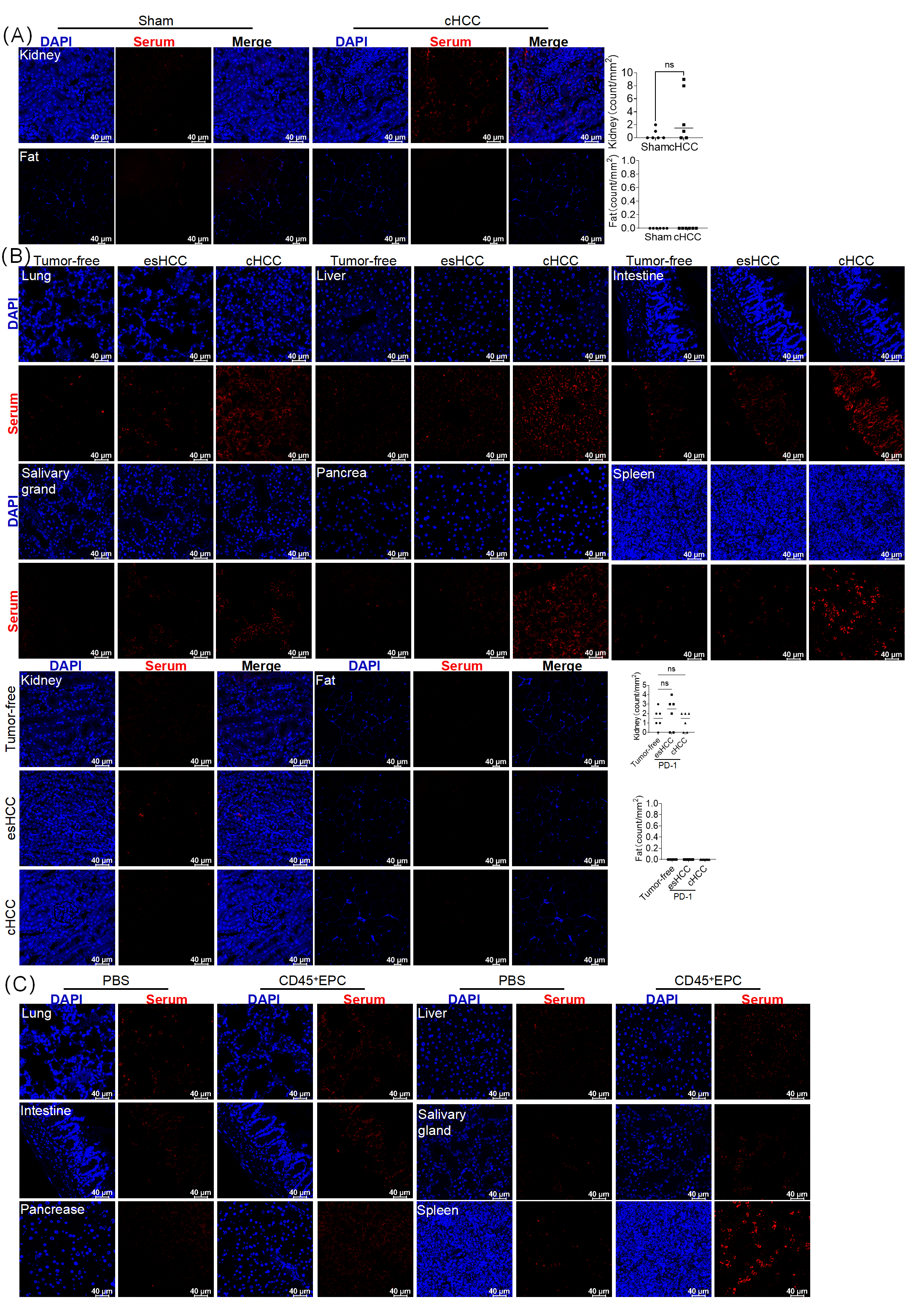

Supplement: Supplementary file 8 — Figure S8. Immunofluorescence supplementation results in Figure 6. (A) Immunofluorescence analysis of autoantibodies combination on kidney and fat sections and statistical results in Rag1−/− mice incubated with serum from sham or cachexic HCC mice. (B) Representative immunofluorescence images of multiple organ sections from Rag1−/− mice in Figure 5D. Statistical results of kidney and fact sections are shown. (C) Representative immunofluorescence images of multiple organ sections from Rag1−/− mice in Figure 5E. [file JCSM-16-e13874-s006.tif]
