# Supplementary material for: Thymic Microenvironment Remodeling in Cancer Cachexia as a Determinant of Checkpoint Inhibitor Efficacy and Toxicity
Source: J Cachexia Sarcopenia Muscle. 2025 Jul 16;16(4):e13874. doi: 10.1002/jcsm.13874 (PMC12264390; doi:10.1002/jcsm.13874)
Supplement: Supplementary file 9 — Table S1. Marker genes of various cell types. Table S2. Gene ontology analysis of the top 100 marker genes of mature mFbs. Table S3. Gene ontology analysis of the top 100 marker genes of immature mFbs. Table S4. Gene ontology analysis of the top 100 marker genes of mFbs from sham mice. Table S5. Gene ontology analysis of the top 100 marker genes of mFbs from cachexic HCC mice. Table S6. Tissue‐specific expression of marker genes (COSG score ≥ 0.6) of mature mFbs. Table S7. Distribution of colontype abundances in TCR‐β repertoire. Table S8. Distribution of colontype abundances in TCR‐α repertoire. Table S9. Inflammatory diseases associated with V/J genes. Table S10. Gene Ontology analysis of DPres subtypes. Table S11. Clinical characteristics of patients with advanced or locally advanced cancer treated with PD‐1/L1 antibody. Table S12. Factors affecting disease progression by univariate Cox regression analysis. Table S13. REAGENT or RESOURC. [file JCSM-16-e13874-s009.docx]

Supplementary materials

Supplementary methods

Cell culture

Hepa1-6 cell lines (ATCC CRL-1830), purchased from the American Type Culture Collection (ATCC), were cultured in dulbecco's modified eagle medium (DMEM) media (Gibco, Thermo Fisher Scientific, Beijing, China) supplemented with 1% penicillin-streptomycin (Gibco) and 10% fetal bovine serum (Gibco) and grown at 37 °C with 5% CO_2_.

Hematoxylin and eosin (HE) staining

Mouse thymuses were isolated, fixed in 4% paraformaldehyde, embedded in paraffin, sectioned, and stained with HE. Pannoramic MIDI (Budapest, Hungary) was used to acquire the images.

Flow cytometry analysis

Thymi were isolated, cut into small pieces, and then digested using 0.25 mg/mL papain (Sigma-Aldrich, USA), 0.25 mg/mL collagenase IV (Sigma-Aldrich, USA), and 0.1 mg/mL DNaseI (Roche Life Science) at 37 ℃ away from light for 30min. Digestion was terminated by adding precool 1×PBS. Then, the mixed liquid was then resuspended and strained, through a 40 mm cell strainer (Biosciences, BD) to obtain uniform cell suspensions. The cells were stained with antibodies (Table S13). For intracellular staining, the FoxP3/Transcription Factor Staining Buffer Kit (MultiSciences Biotech Co., Ltd, Hangzhou, China) was used following the manufacturer’s instructions. CytoFLEXLX (Beckman Coulter Life Science) was used to evaluate cell phenotypes by collecting data as the fraction of labeled cells with at least 50,000 events. CytoFLEX SRT was used for the flow cytometric sorting. FlowJo V10.0.7 (FlowJo, OR, USA) and CytExpert (2.4.0.28) were used to analyze the data.

Immunofluorescence analysis

The paraffin-embedded tissue sections were first rehydrated using xylene, gradient ethanol, and deionized H2O. Next, 3% hydrogen peroxide was used to block endogenous catalase under the condition of microwave heating at low power for 3 min. Antigen retrieval was performed by microwaving the sections in 10 mM citric acid for 2 min at high power, followed by 5 min at 50% power. After cooling for approximately 60 min at 25 ℃, 0.5% Bovine Serum Albumin (BSA) was used to block non-specific staining for 30 min at 37 ℃. Sections were incubated with primary antibodies (Table S13) overnight at 4 ℃ and then incubated with 1:400 diluted goat anti-rat Alexa Fluor 488 (Cat#ab150165, Abcam, Cambridge, MA), goat anti-rabbit Alexa Fluor 594 (Cat#ab150088, Abcam, Cambridge, MA), goat anti-mouse Alexa Fluor 647 (Cat#ab150119, Abcam, Cambridge, MA), goat anti-mouse Alexa Fluor 594 (Cat#ab150116, Abcam, Cambridge, MA) or goat anti-human IgG (H+L) Alexa Fluor 488 (A-11013, Thermo) secondary antibodies for 45 min at 37℃. Finally, DAPI (Mounting Medium with DAPI-Aqueous, Fluor Shield Abcam, Cambridge, MA, USA; ab104139) was used to stain the nuclei. Fluorescent signals were detected using Leica STELLARIS STED, ECLIPSE Ni-U, or ECLIPSE Ti2-U. Images of the tissues were analyzed by using Leica Application Suite X 1.8.0.13313 or NIS Viewer (5.21.00). The absolute number of positive cells/mm2 in nine fields of interest was statistically analyzed.

Enzyme-linked immunosorbent assay (ELISA)

Serum samples were collected from the cachexic HCC and sham mice. The concentrations of Mmp9 and Hmgcs2 antibodies in the sera were measured using ELISA (Mice Mmp9 Ab ELISA Kit, MM-47001M1; Mice Hmgcs2 Ab ELISA Kit, MM-46994M1, MEIMIAN) following the manufacturer’s instructions.

Preparation of single-cell suspensions for single-cell RNA sequencing

Mouse thymuses were isolated and transferred to DMEM/F-12 medium (C11330500BT, Thermo Fisher Scientific) containing 10% fetal tissue on ice. The thymuses were then digested into single-cell suspensions using the process described above. CD45^-^ thymus cells were collected using the EasySep™ Mouse CD45 Positive Selection Kit (Stemcell, Vancouver, Canada).

Single-cell RNA sequencing and single-cell TCR sequencing

scRNA-seq was performed using a chromium single-cell platform (10xGenomic, Pleasanto, CA, USA) according to the manufacturer’s instructions. Cellular suspensions were encapsulated into single-cell Gel Bead-In- EMulsions (GEMs), followed by reverse transcription and cDNA library construction using either Single Cell 5’ Reagent or 3’ Reagent Kit, and sequenced using the Illumina platform. The original data underwent filtering for quality control, compared, quantified, identified, and recycled, the gene expression matrix of each cell was obtained, using Cell Ranger Software (version 6.0.1; 10× Genomics). TCRαβ paired VDJ libraries were prepared from samples using the 5’ Reagent kit. Single-cell TCR sequencing was performed and quantified using the Cellranger-vdj software (version 6.0.1).

Data processing

Data were analyzed by using the Seurat package (version 4.4.0) in R (version 4.2.2). Cells with fewer than 300 detected genes, less than 500 UMI, greater than 30000 UMI, or total mitochondrial gene expression exceeding 10% were excluded. Genes that were expressed in less than ten cells were also excluded. Individual datasets were merged, and the harmony package (1.0.1) was used to remove the batch effect if necessary. After quality control, data analysis was performed through normalization, feature selection, data scaling, linear dimensional reduction, dimensionality determination, cell clustering, and non-linear dimensional reduction (UMAP/tSNE). Cell annotation was based on cell-type marker genes reported in other research (Table S1) and the results of the correlation analysis with bulk RNA-seq data (GSE15907). After cell annotation, specific cell types were extracted for subtype analysis using the process described above.

Functional enrichment analysis

The COSG package (version 0.9.0) was used to identify the marker genes of thymus medullary fibroblasts in mature and immature subtypes, and thymus medullary fibroblasts in sham and cachexic HCC mice. The top 100 genes were used for GO/KEGG/ReactomePA analyses using the ClusterProfiler package (4.6.2). The FindAllMarkers function (Seurat, version 4.4.0) was used to identify the marker genes of DPres1, DPres2, and DPres3. The top 50 genes were used for GO analysis.

Cell-Cell Communication Analysis

Mature and immature thymus medullary fibroblasts and CD4/CD8 single-positive thymocytes were extracted for the inference and analysis of cell-cell communication using the cellChat package (version 1.6.1, http://www.cellchat.org/), a public repository of ligands, receptors, cofactors, and their interactions.

Cell trajectory analysis

Raw count data from DPsels cells were used to construct a trajectory in pseudotime using the monocle2 package (version 2.26.0). Internal normalization was conducted by estimating the size factors. Differential gene expression analysis was performed using the DifferentialGeneTest function, with the top 945 genes used to build a cell trajectory of cells using the DDRTree algorithm.

TCR repertoire data analysis

TCR repertoire analysis was performed using the Immunarch package (version 1.0.0). The Immunarch package (1.0.0) was used for TCR repertoire scrutinization including clonotype profiling, diversity (Chao1 value, and rarefaction analysis), CDR3 length, and V/J gene usage.

Definition of Tissue-restricted genes

Marker genes (cosg score ≥0.6) of mature thymus medullary fibroblasts determined using COSG package (version 0.9.0) and scrutinized for tissue-restricted genes using TiGER（http://bioinfo.wilmer.jhu.edu/tiger/）

Study participants

Patients with advanced cancer receiving anti-PD-1/L1 antibody-containing treatment at the Third Affiliated Hospital of Sun Yat-sen University between June 1, 2020, and October 31, 2023, were eligible. The inclusion criteria were as follows :(1) age≥18 years but <80 years, (2) pathological diagnosis of advanced malignant tumor, and (3) clinical indication for anti-PD-1/L1 antibody treatment. The exclusion criteria were as follows: (1) mental illness, (2) refusal or inability to adhere to the treatment, and (3) pregnancy or lactation.

Statistical analysis

Data analysis was performed using GraphPad Prism 9 software (GraphPad Software, La Jolla, CA, USA). For two population comparisons, the student’s t-test or Mann–Whitney U-test was performed based on the normality of the distribution. The Friedmann test and one-way ANOVA were used for comparisons involving more than two groups. MedCalc (version 22.001) was used for ROC and survival analysis. The autoReg (version 0.3.2) R package was used for univariate Cox regression analysis. A *P*-value <0.05 was considered significant.

Supplementary figures


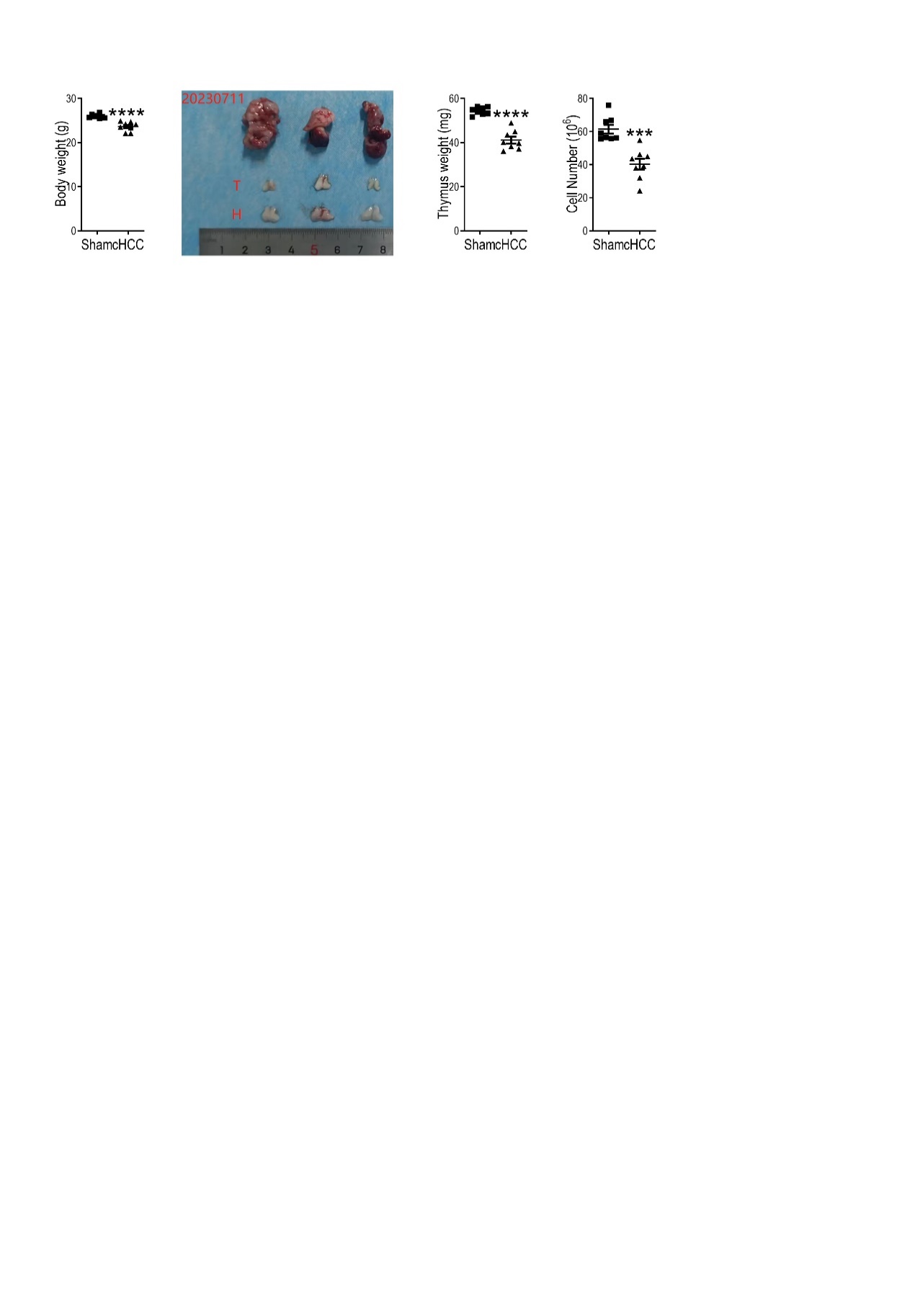


Figure S1 Body weights, tumor burden, thymus weight and cell number of sham (n=8) and cachexic HCC (n=8) mice.


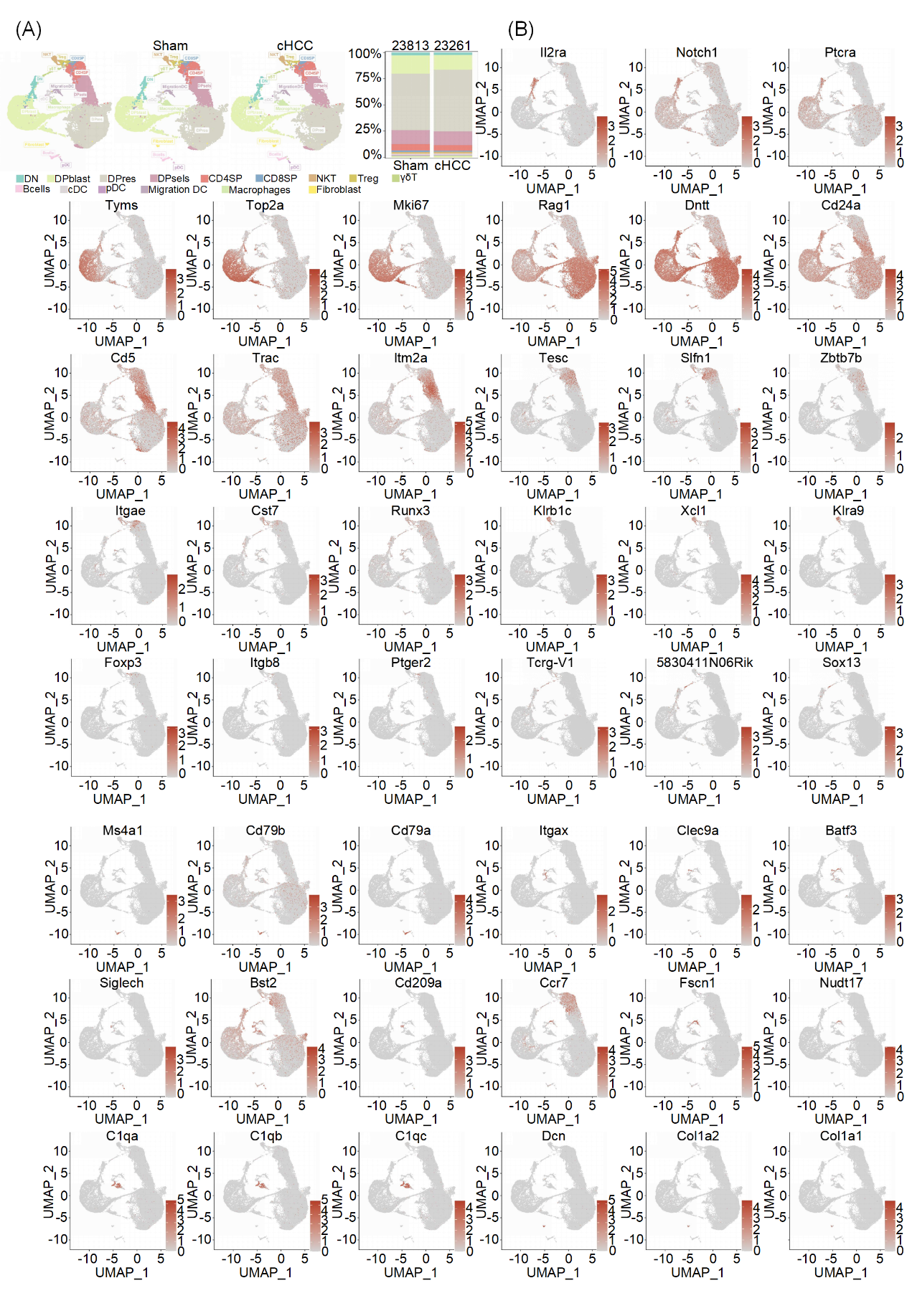


**Figure S2.** **Identification of cell types. (A)** Two-dimensional representation of cells and cells split by group via umap and ratio of cell types in each group via bar chart, which were colored based on cell type identity in the whole thymocytes. **(B)** marker genes of DN, DPblast, DPres, DPsels, CD4SP, CD8SP, NKT, Treg, γδT, B cells, cDC, pDC, migration DC, macrophages, and fibroblasts projected onto umap plots.


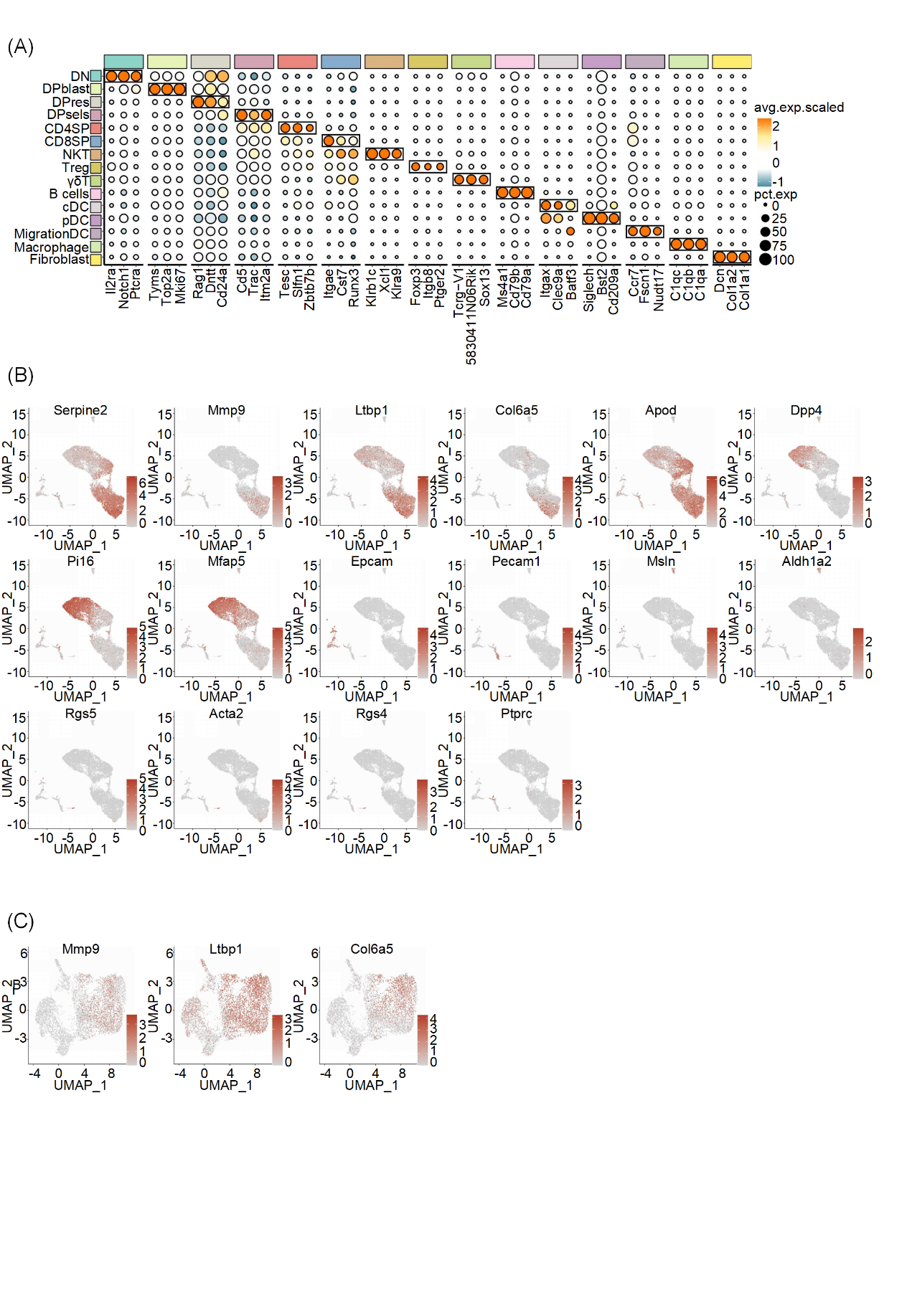


**Figure S3.** **Identification of cell types (A)** Dot plot displaying marker genes of DN, DPblast, DPres, DPsels, CD4SP, CD8SP, NKT, Treg, γδT, B cells, cDC, pDC, migration DC, macrophages, and fibroblasts. **(B-C)** Marker genes of capFb, mFb, TEC, endothelial cells, Mesothelial cells, pericytes, immune cells (B), immature mFbs, and mature mFbs (C) projected onto umap plots.


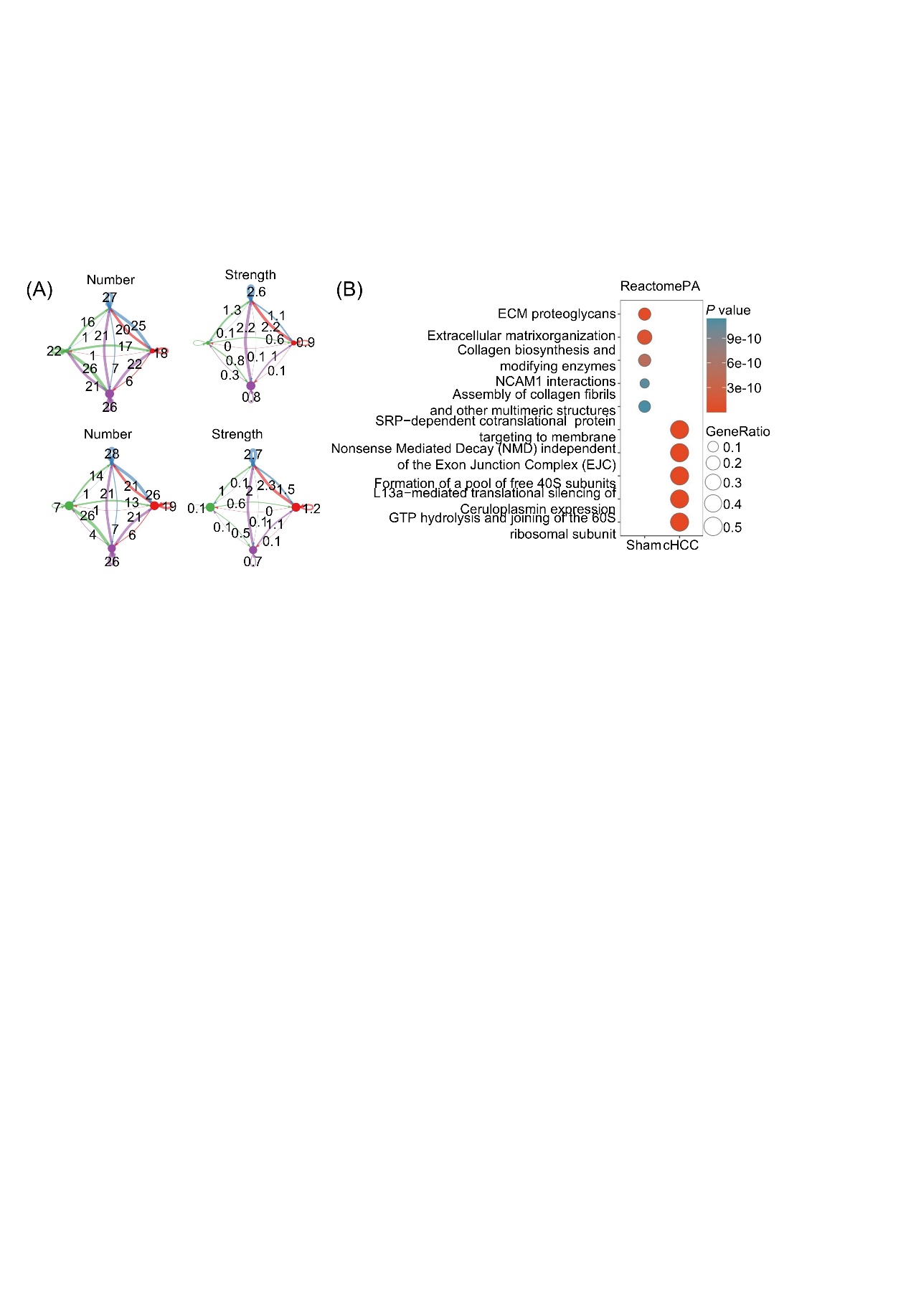


Figure S4 (A) Interaction analysis among CD4SP, CD8SP, immature mFbs and mature mFbs in sham and cachexic HCC mice by the cellChat package (version 1.6.1) in R. Number and weight of cell-cell interactions. (B) ReactomePA analysis of mFbs of the thymus in sham mice and cachexic HCC mice. Items associated with antigen processing and presentation functions are marked in red.


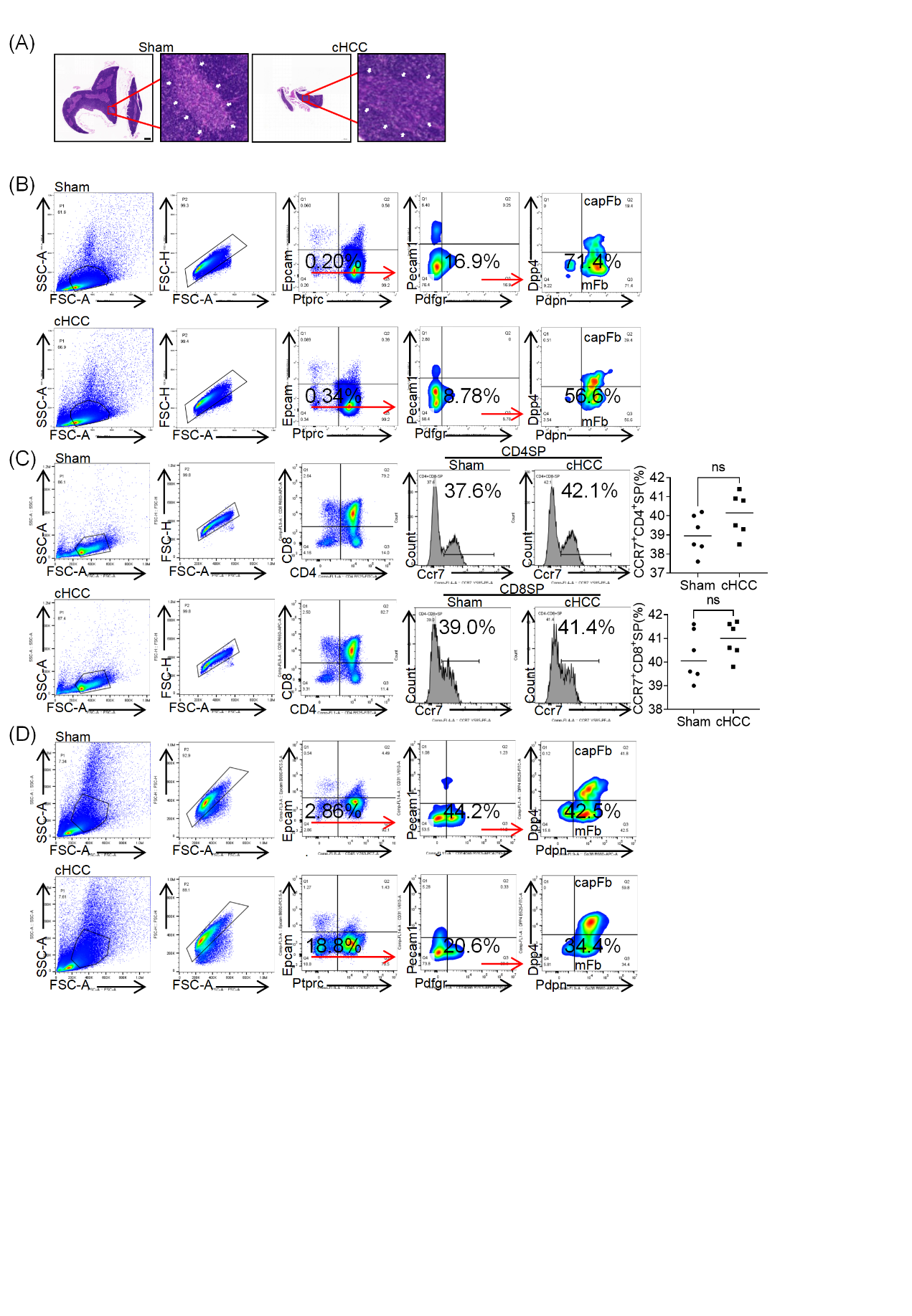


**Figure S**5**. Supplementary results in Figure 2. (A)** Representative hematoxylin-eosin (H&E) staining images showing changes in the thymic medulla in cachexic HCC and sham mice. White arrows indicate the corticomedullary junction. **(B)** Sequential gating strategy for Mmp9^+^mFbs identification. **(C)** Flow cytometry analysis of the expression of CCR7 on CD4/8 SP thymocytes in sham and cachexic HCC mice. **(D)** Sequential gating strategy for Ccl19^+^mFbs identification.


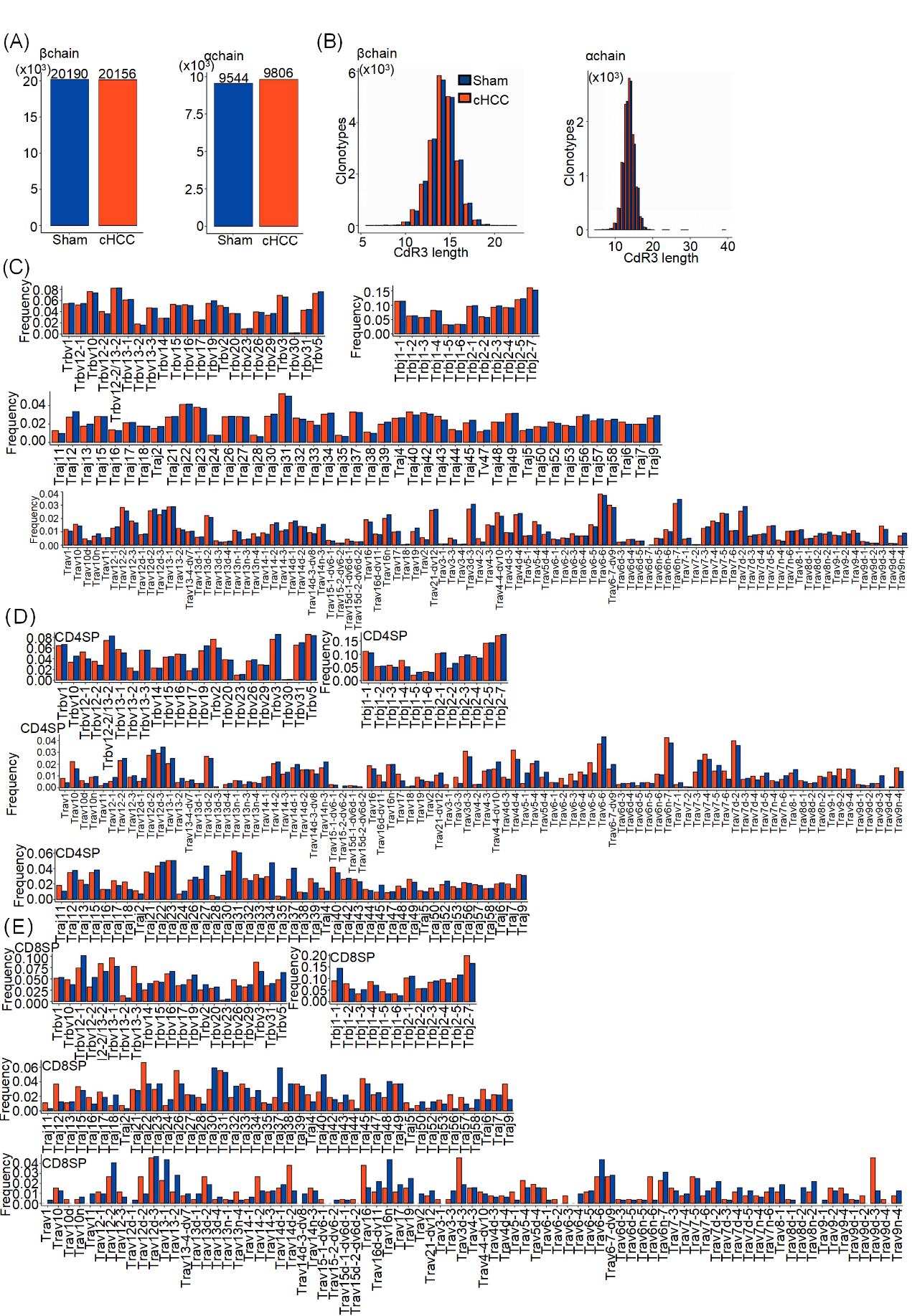


**Figure S**6**. Usage of v/j genes of thymocytes**. **(A)** The number of unique clonotypes of TCR-β and TCR-α repertoires in thymocytes of cachexic HCC and sham mice. **(B)** CDR3 length distribution of the β and α chains in the thymocytes of cachexic HCC and sham mice. **(C)** Comparison of the usage of v/j gene of the β and α chains in the thymocytes of cachexic HCC and sham mice. **(D)** Comparison of the usage of v/j genes of the β and α chains in CD4SP thymocytes of cachexic HCC and sham mice. **(E)** Comparison of the usage of v/j gene of the β and α chains in CD8SP thymocytes of cachexic HCC and sham mice. The analysis was performed by using the Immunarch package (1.0.0) in R.


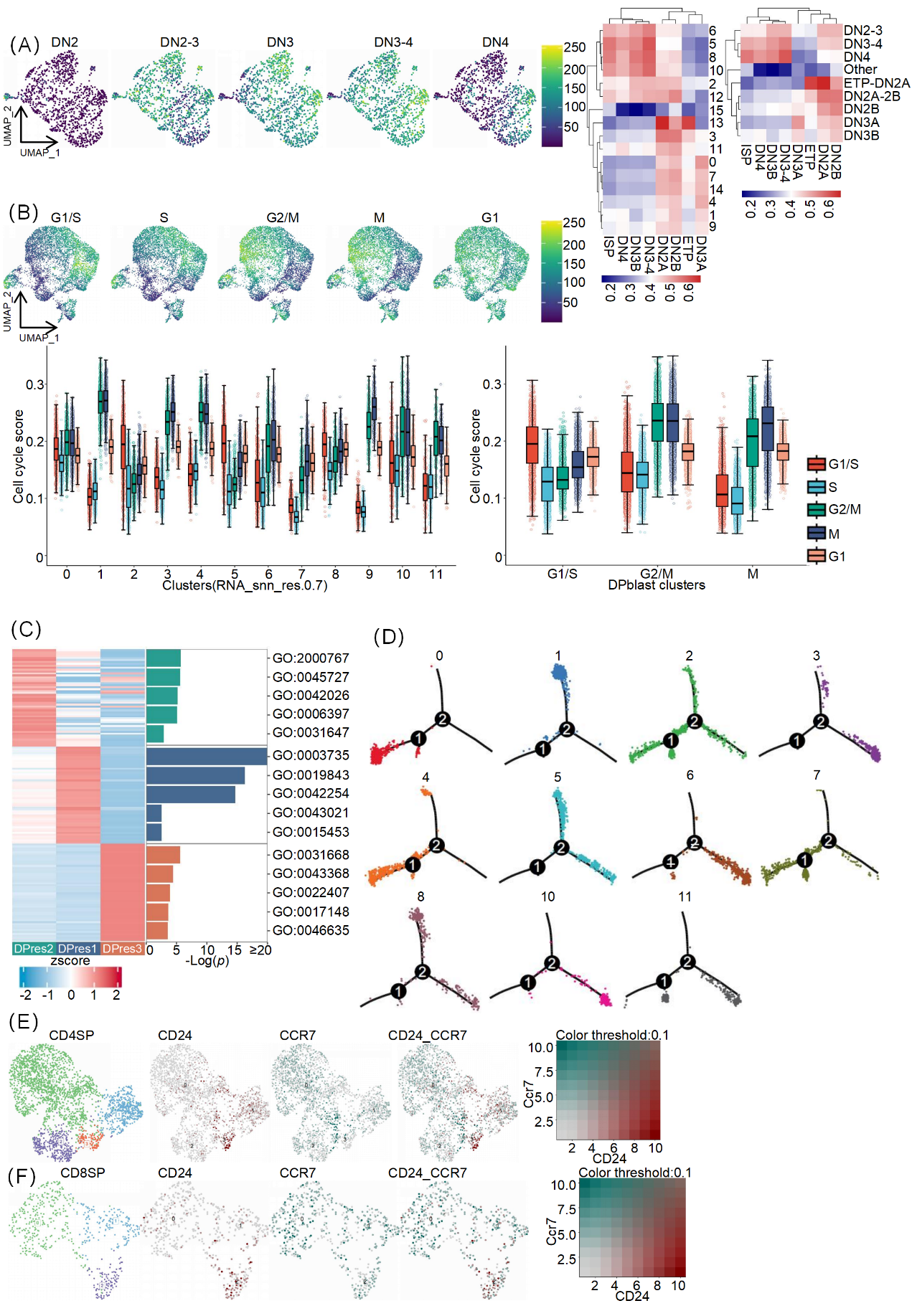


**Figure S**7**. Thymocyte subtypes analysis**. **(A)** Two-dimensional representation (via umap) of the score of marker genes associated with the differential stage of DN thymocytes using the AUC package (version 1.20.2). Heatmaps show correlation (via psych package, version 2.3.9) of gene expression profiles between bulk-sorted thymocyte subpopulations (GSE15907) and our single-cell clusters (resolution.1.7, left) and annotated thymocyte types (right), respectively. **(B)** Two-dimensional representation (via umap) of the score of marker genes of cell cycles using the AUC package (1.20.2). Comparisons of cell cycle score using the AUC package (1.20.2) in different single-cell clusters (resolution.0.7, left) and different annotated DPblasts subtypes (right), respectively. **(C)** Gene ontology analysis of the top 50 marker genes from each DPres thymocyte subtypes using FindAllMarkers functions in the Seurat (4.4.0) and clusterProfiler (4.6.2) packages. **(D)** Distribution of each cluster (resolution.1.0) of DPsels thymocytes in trajectory via trajectory analysis by monocle (2.26.2) package. Cluster 9 was deleted for belonging to contaminated cells. **(E-F)** Two-dimensional representation via umap of clusters and expression of CD24 and CCR7 in CD4SP (up) and CD8SP (down) thymocytes.


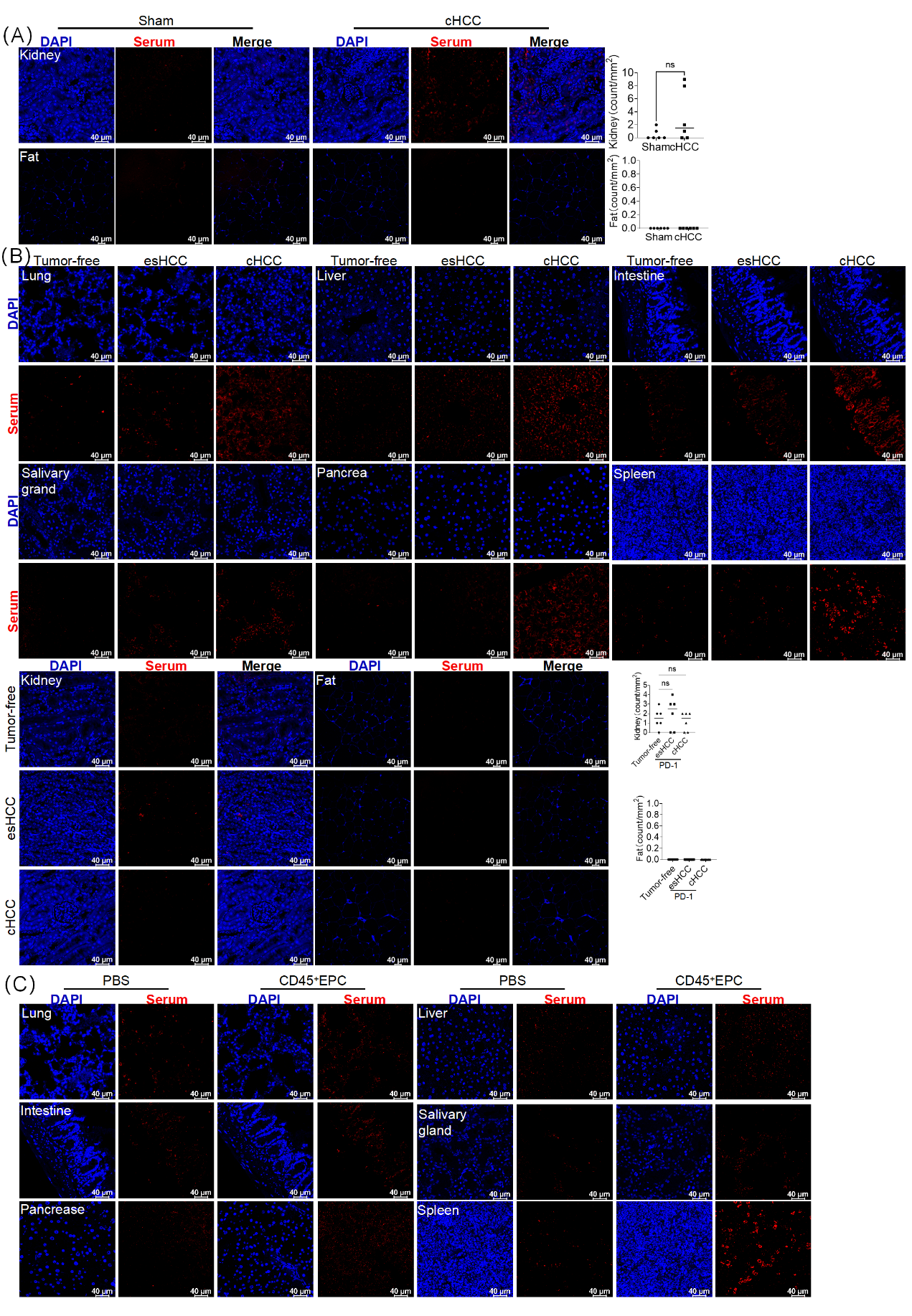


**Figure S**8**.** **Immunofluorescence supplementation results in Figure 6** **(A)** Immunofluorescence analysis of autoantibodies combination on kidney and fat sections and statistical results in Rag1-/- mice incubated with serum from sham or cachexic HCC mice. **(B)** Representative immunofluorescence images of multiple organ sections from Rag1^-/-^ mice in Figure 5D. statistical results of kidney and fact sections are shown. **(C)** Representative immunofluorescence images of multiple organ sections from Rag1^-/-^ mice in Figure 5E.

Supplementary Tables

Table S1. Marker genes of various cell types

| Cell types | Markers | PMID |
| --- | --- | --- |
| DN | Il2ra | 33771202 |
|  | Notch1 | 33771202 |
|  | Ptcra | 33771202 |
| DP blasts | Tyms | 33771202 |
|  | Top2a | 33771202 |
|  | Mki67 | 33771202 |
| DPres | Rag1 | 33397934 |
|  | Dntt | 33397934 |
|  | Cd24a | 33397934 |
| DPsels | Cd5 | 33771202 |
|  | Trac | 33771202 |
|  | Itm2a | 33771202 |
| CD4SP | Tesc | 37580604 |
|  | Slfn1 | 37580604 |
|  | Zbtb7b | 33397934 |
| CD8SP | Itgae | 33397934 |
|  | Cst7 | 33397934 |
|  | Runx3 | 33397934 |
| NKT | Klrb1c | 35915069 |
|  | Xcl1 | 15345586 |
|  | Klra9 | 33173202 |
| Treg | Foxp3 | 36741366 |
|  | Itgb8 | 29109269 |
|  | Ptger2 | 19536194 |
| γδT | Tcrg-V1 | 37580604 |
|  | 5830411N06Rik | 36926655 |
|  | Sox13 | 32627520 |
| Bcells | Ms4a1 | 34083450 |
|  | Cd79b | 36420662 |
|  | Cd79a | 36420662 |
| cDC | Itgax | 32433953 |
|  | Clec9a | 36420662 |
|  | Batf3 | 32433953 |
| pDC | Siglech | 31467405 |
|  | Bst2 | 31467405 |
|  | Cd209a | 31467405 |
| mDC | Ccr7 | 36685557 |
|  | Fscn1 | 36685557 |
|  | Ccl5 | 32392463 |
| Macrophages | C1qc | 36420662 |
|  | C1qb | 36420662 |
|  | C1qa | 36420662 |
| Fibroblasts | Dcn | 36420662 |
|  | Col1a2 | 36420662 |
|  | Col1a1 | 36420662 |
| mFb | Serpine | 34096078 |
| mature mFb | Mmp9 | 34096078 |
|  | Ltbp1 | 34096078 |
|  | Col6a5 | 34096078 |
| Immature/mature mFb | Apod | 34096078 |
| CapFb | Dpp4 | 34096078 |
|  | Pi16 | 34096078 |
|  | Mfap5 | 34096078 |
| Endothelial cell | Pecam1 | 34096078 |
| Mesothelial cell | Msln | 34096078 |
|  | Aldh1a2 | 34096078 |
| TEC | Epcam | 34096078 |
| Pericytes | Rgs5 | 37350296 |
|  | Acta2 | 35262195 |
|  | Rgs4 | 37350296 |
| Immune cells | Ptprc | 36495148 |

DN, double-negative; DP, double-positive; DPblast, double-positive blast thymocytes; DPres, double-positive thymocytes undergoing rearrangement; DPsels, double positive thymocytes undergoing selection; SP, single-positive; NKT, natural killer T cell; Treg, regulatory T cell; cDC, conventional dendritic cell; pDC, plasmacytoid dendritic cell; mDC, migration dendritic cell; mFb, medullary fibroblast; capFb, capsular fibroblast; TEC, thymic epithelial cell.

Table S2. Gene ontology analysis of the top 100 marker genes of mature mFbs

| Category | ID | Description |
| --- | --- | --- |
| BP | GO:0002428 | Antigen processing and presentation of peptide antigen via MHC class Ib |
|  | GO:0002483 | Antigen processing and presentation of endogenous peptide antigen |
|  | GO:0001916 | Positive regulation of T cell-mediated cytotoxicity |
|  | GO:0030198 | Extracellular matrix organization |
|  | GO:0031589 | Cell-substrate adhesion |
| CC | GO:0062023 | Collagen-containing extracellular matrix |
|  | GO:0042612 | MHC class I protein complex |
|  | GO:0005581 | Collagen trimer |
|  | GO:0033106 | Cis-Golgi network membrane |
|  | GO:0031594 | Neuromuscular junction |
| MF | GO:0005201 | Extracellular matrix structural constituent |
|  | GO:0005178 | Integrin binding |
|  | GO:0046979 | TAP2 binding |
|  | GO:0046978 | TAP1 binding |
|  | GO:0005518 | Collagen binding |

BP, biological process; CC, cellular component; MF, molecular function.

Table S3. Gene ontology analysis of the top 100 marker genes of immature mFbs

| Category | ID | Description |
| --- | --- | --- |
| BP | GO:0002181 | Cytoplasmic translation |
|  | GO:0006749 | Glutathione metabolic process |
|  | GO:0001503 | Ossification |
|  | GO:0048144 | Fibroblast proliferation |
|  | GO:0061138 | Morphogenesis of a branching epithelium |
| CC | GO:0062023 | Collagen-containing extracellular matrix |
|  | GO:0022626 | Cytosolic ribosome |
|  | GO:0098800 | Inner mitochondrial membrane protein complex |
|  | GO:0031091 | Platelet alpha granule |
|  | GO:0019867 | Outer membrane |
| MF | GO:1901681 | Sulfur compound binding |
|  | GO:0003735 | Structural constituent of ribosome |
|  | GO:0008083 | Growth factor activity |
|  | GO:0008237 | Metallopeptidase activity |
|  | GO:0004601 | Peroxidase activity |

BP, biological process; CC, cellular component; MF, molecular function.

Table S4. Gene ontology analysis of the top 100 marker genes of mFbs from sham mice

| Category | ID | Description |
| --- | --- | --- |
| BP | GO:0042026 | Protein refolding |
|  | GO:0030198 | Extracellular matrix organization |
|  | GO:0043062 | Extracellular structure organization |
|  | GO:0002474 | Antigen processing and presentation of peptide antigen via MHC class I |
|  | GO:0002501 | Peptide antigen assembly with MHC protein complex |
| CC | GO:0062023 | Collagen-containing extracellular matrix |
|  | GO:0005581 | Collagen trimer |
|  | GO:0042612 | MHC class I protein complex |
|  | GO:0030670 | Phagocytic vesicle membrane |
|  | GO:0042824 | MHC class I peptide loading complex |
| MF | GO:0005201 | Extracellular matrix structural constituent |
|  | GO:0051082 | Unfolded protein binding |
|  | GO:0044183 | Protein folding chaperone |
|  | GO:0005518 | Collagen binding |
|  | GO:0042288 | MHC class I protein binding |

BP, biological process; CC, cellular component; MF, molecular function.

Table S5. Gene ontology analysis of the top 100 marker genes of mFbs from cachexic HCC mice

| Category | ID | Description |
| --- | --- | --- |
| BP | GO:0002181 | Cytoplasmic translation |
|  | GO:0042254 | Ribosome biogenesis |
|  | GO:1901798 | Positive regulation of signal transduction by p53 class mediator |
|  | GO:0006749 | Glutathione metabolic process |
|  | GO:0140694 | Non-membrane-bounded organelle assembly |
| CC | GO:0062023 | Collagen-containing extracellular matrix |
|  | GO:0005751 | Mitochondrial respiratory chain complex IV |
|  | GO:0043209 | Myelin sheath |
|  | GO:0098984 | Neuron to neuron synapse |
|  | GO:0001726 | Ruffle |
| MF | GO:0003735 | Structural constituent of ribosome |
|  | GO:0019843 | rRNA binding |
|  | GO:1990948 | Ubiquitin ligase inhibitor activity |
|  | GO:1901681 | Sulfur compound binding |
|  | GO:0016209 | Antioxidant activity |

BP, biological process; CC, cellular component; MF, molecular function.

Table S6. Tissue-specific expression of marker genes (COSG score≥0.6) of mature mFbs

| Genes | Score | Tissue-specific expression |
| --- | --- | --- |
| Serpine2 | 0.766858906 | No |
| Enpp2 | 0.757291187 | No |
| Ltbp1 | 0.750914331 | Bone |
| Des | 0.740782416 | Heart, larynx, muscle |
| Mfge8 | 0.740487018 | No |
| Ltbp4 | 0.733897276 | No |
| Postn | 0.733627028 | Heart, tongue |
| H2-D1 | 0.724811294 | Lung, thymus |
| Hspa1a | 0.72194633 | Peripheral nervous system, soft tissue |
| Hspa1b | 0.721239451 | Bladder |
| H2-K1 | 0.720798287 | No |
| Ltc4s | 0.70421774 | No |
| Ifitm1 | 0.698619038 | Bone marrow |
| Myh9 | 0.69061314 | Larynx |
| Lars2 | 0.685426474 | No |
| Col6a2 | 0.670490359 | Bone |
| mt-Atp8 | 0.669463709 | No |
| Malat1 | 0.668732451 | No |
| Ly6e | 0.666955834 | No |
| Col15a1 | 0.665008677 | No |
| H2-Q7 | 0.664590226 | No |
| Sptbn1 | 0.660758056 | Tongue |
| Il34 | 0.659154103 | No |
| Gm42418 | 0.65782302 | No |
| B2m | 0.654233292 | Blood |
| Lamb1 | 0.654000766 | Soft tissue |
| Zbtb20 | 0.651990031 | No |
| Ddx5 | 0.651048186 | No |
| Ptn | 0.647566796 | No |
| mt-Co3 | 0.646454247 | No |
| Bgn | 0.645574752 | Bone, spleen |
| mt-Atp6 | 0.64223631 | No |
| C3 | 0.639222926 | Liver |
| Mylk | 0.637738137 | No |
| Ahnak | 0.636596897 | No |
| Sod3 | 0.636072405 | Peripheral nervous system, soft tissue |
| H2-T23 | 0.62930922 | No |
| AY036118 | 0.628463467 | No |
| Marcks | 0.624949322 | No |
| Hspa5 | 0.624213709 | No |
| Ifngr1 | 0.621611517 | Soft tissue |
| Ndufa4l2 | 0.620536787 | No |
| Igfbp7 | 0.619626894 | larynx |
| Son | 0.617118289 | No |
| Cald1 | 0.616852769 | Spleen |
| Tcf4 | 0.616451504 | Tongue |
| Lhfp | 0.616339477 | No |
| Ptma | 0.612553929 | No |
| Flt3l | 0.611523485 | Eye |
| mt-Co2 | 0.610309766 | No |
| mt-Cytb | 0.609505025 | No |
| mt-Co1 | 0.60900342 | No |
| Gm26917 | 0.60793481 | No |
| Nrp1 | 0.602415393 | No |
| mt-Nd4l | 0.601432775 | No |
| H2-Q4 | 0.600751808 | No |

Table S7. Distribution of colontype abundances in TCR-β repertoire

| Sample | Clone.num | Clonotypes |
| --- | --- | --- |
| Sham | 1 | 19600 |
|  | 2 | 515 |
|  | 3 | 54 |
|  | 4 | 11 |
|  | 5 | 6 |
|  | 6 | 2 |
|  | 7 | 1 |
|  | 18 | 1 |
|  |  |  |
| cHCC | 1 | 19455 |
|  | 2 | 600 |
|  | 3 | 71 |
|  | 4 | 19 |
|  | 5 | 9 |
|  | 6 | 2 |
|  | / | / |
|  | / | / |

cHCC, cachexic hepatocellular carcinoma.

Table S8. Distribution of colonotype abundances in TCR-α repertoire

| Sample | Clone.num | Clonotypes |
| --- | --- | --- |
| Sham | 1 | 9216 |
|  | 2 | 263 |
|  | 3 | 45 |
|  | 4 | 10 |
|  | 5 | 4 |
|  | 7 | 21 |
|  | 8 | 1 |
|  | 20 | 1 |
|  | 38 | 1 |
| cHCC | 46 | 1 |
|  | 68 | 1 |
|  |  |  |
|  | 1 | 9391 |
|  | 2 | 344 |
|  | 3 | 43 |
|  | 4 | 18 |
|  | 5 | 3 |

cHCC, cachexic hepatocellular carcinoma.

Table S9. Inflammatory diseases associated V/J genes

| V/Jgenes | Autoimmune disease | PMID |
| --- | --- | --- |
| Trbv1 | Reactive arthritis | 27869234 |
|  | Type I diabetes disease | 24146886/27161799 |
|  | Myeloperoxidase induced autoimmunity | 28318808 |
| Trbv2 |  |  |
| Trbv3 | Sjögren's syndrome | 29679709 |
|  | Aire-deficiency | 28318808 |
| Trbv10 |  |  |
| Trbv13-2 | myelin oligodendrocyte glycoprotein-induced experimental autoimmune encephalomyelitis | 27869234/28318808 |
|  | Type I diabetes disease | 27161799 |
| Trbv13-3 | Type I diabetes disease | 24146886/27161799/35667687 |
|  | Myeloperoxidase induced autoimmunity | 28318808 |
|  | Dry eye | 36877515 |
| Trbv15 | Type I diabetes disease | 19917704 |
|  | Dry eye | 36877515 |
| Trbv16 | Sjögren's syndrome | 28970488 |
| Trbv17 | Aire-defeciency | 28318808 |
| Trbv23 | Sjögren's syndrome | 28970488 |
| Trbv26 |  |  |
| Trbv31 | Atherosclerosis | 20439543 |
| Trbj1-1 | Type I diabetes disease | 27161799 |
| Trbj1-4 |  |  |
| Trbj1-5 |  |  |
| Trbj1-6 | myeloperoxidase induced autoimmunity | 28318808 |
| Trbj2-2 | Sjögren's syndrome | 28970488 |
|  | Type I diabetes diseases | 35667687 |
| Trbj2-7 | Type I diabetes disease | 27161799/35667687 |
| Trav1 |  |  |
| Trav10 | Type 1 Diabetes | 34868047 |
| Trav10d |  |  |
| Trav12-2 | Primary biliary cholangitis | 30690835 |
| Trav12d-1 |  |  |
| Trav12d-2 |  |  |
| Trav12d-3 |  |  |
| Trav13-2 | Primary Sjögren's Syndrome | 33603736/28970488 |
| Trav13d-1 | Sjögren's syndrome | 28970488 |
| Trav14d-3-dv8 |  |  |
| Trav15-1-dv6-1 |  |  |
| Trav16n | Type 1 Diabetes | 34868047/35667687 |
| Trav17 | Type 1 Diabetes | 35626661 |
| Trav21-dv12 |  |  |
| Trav4-2 |  |  |
| Trav4-4-dv10 |  |  |
| Trav4d-3 |  |  |
| Trav4d-4 |  |  |
| Trav5-1 |  |  |
| Trav5-4 | Type 1 Diabetes | 34868047 |
| Trav6-1 |  |  |
| Trav6-3 |  |  |
| Trav6-4 | Dry eye | 36877515 |
| Trav6-5 |  |  |
| Trav6n-6 |  |  |
| Trav7-1 |  |  |
| Trav7-3 |  |  |
| Trav7-4 | Dry eye | 36877515 |
| Trav7d-3 |  |  |
| Trav7d-5 |  |  |
| Trav7n-6 |  |  |
| Trav8-1 | Type 1 diabetes | 26953160 |
|  | Sjögren's syndrome | 28970488 |
| Trav9-1 |  |  |
| Trav9-4 |  |  |
| Trav9d-1 |  |  |
| Trbj12 |  |  |
| Traj15 | Dry eye |  |
| Traj17 | Atherosclerosis | 36684560 |
| Traj18 |  |  |
| Traj22 |  |  |
| Traj24 |  |  |
| Traj31 |  |  |
| Traj33 | Autoimmune pancreatitis | 36376329 |
| Traj40 |  |  |
| Traj42 | Type 1 Diabetes | 34868047/35667687 |
| Traj48 |  |  |
| Traj52 | Sjögren's syndrome | 28970488 |
| Traj53 | Type 1 Diabetes | 34868047 |
| Traj56 |  |  |
| Traj57 |  |  |
| Traj58 |  |  |
| Traj9 | Type 1 diabetes | 26953160 |

Table S10. Gene Ontology analysis of DPres subtypes

| Subtypes | ID | Description |
| --- | --- | --- |
| DPres2 | GO:2000767 | Positive regulation of cytoplasmic translation |
|  | GO:0045727 | Positive regulation of translation |
|  | GO:0042026 | Protein refolding |
|  | GO:0006397 | mRNA processing |
|  | GO:0031647 | Regulation of protein stability |
| DPres1 | GO:0003735 | Structural constituent of ribosome |
|  | GO:0019843 | rRNA binding |
|  | GO:0042254 | Ribosome biogenesis |
|  | GO:0043021 | Ribonucleoprotein complex binding |
|  | GO:0015453 | Oxidoreduction-driven active transmembrane transporter activity |
| DPres3 | GO:0031668 | Cellular response to extracellular stimulus |
|  | GO:0043368 | Positive T cell selection |
|  | GO:0022407 | Regulation of cell-cell adhesion |
|  | GO:0017148 | Negative regulation of translation |
|  | GO:0046635 | Positive regulation of alpha-beta T cell activation |

DPres, double positive thymocytes undergoing rearrangement.

Table S11. Clinical characteristics of patients with advanced or locally advanced cancer treated with PD-1/L1 antibody

| Characteristics | level | | Overall | Treatment efficacy | | | | P |
| --- | --- | --- | --- | --- | --- | --- | --- | --- |
|  |  |  |  | CR | PR | SD^*^ | PD |  |
| n |  |  | 65 | 2 | 18 | 21 | 24 |  |
| Sex (%) | Female | | 13 (20.00) | 1 (50.00) | 1 (5.56) | 5 (23.81) | 6 (25.00) | 0.2574 |
|  | Male | | 52 (80.00) | 1 (50.00) | 17 (94.44) | 16 (76.19) | 18 (75.00) |  |
| Age (mean (SD)) |  |  | 58.754 (12.602) | 54.000 (4.243) | 59.611 (10.944) | 57.619 (14.253) | 59.500 (13.102) | 0.8962 |
| Height(m) (mean (SD)) |  |  | 1.650 (0.079) | 1.590 (0.014) | 1.647 (0.062) | 1.652 (0.091) | 1.654 (0.083) | 0.7459 |
| Weight(kg) (mean (SD)) |  |  | 60.346 (11.198) | 60.500 (14.849) | 63.056 (9.625) | 57.143 (11.682) | 61.104 (11.682) | 0.4179 |
| TNM | T(%) | 1 | 7 (10.77) | 0 (0.00) | 3 (16.67) | 2 (9.52) | 2 (8.33) | 0.3941 |
|  |  | 2 | 8 (12.31) | 0 (0.00) | 2 (11.11) | 2 (9.52) | 4 (16.67) |  |
|  |  | 3 | 11 (16.92) | 0 (0.00) | 3 (16.67) | 6 (28.57) | 2 (8.33) |  |
|  |  | 4 | 35 (53.85) | 1 (50.00) | 10 (55.56) | 10 (47.62) | 14 (58.33) |  |
|  |  | x | 4 (6.15) | 1 (50.00) | 0 (0.00) | 1 (4.76) | 2 (8.33) |  |
|  | N(%) | 0 | 12 (18.46) | 1 (50.00) | 2 (11.11) | 5 (23.81) | 4 (16.67) | 0.1545 |
|  |  | 1 | 19 (29.23) | 0 (0.00) | 3 (16.67) | 5 (23.81) | 11 (45.83) |  |
|  |  | 2 | 13 (20.00) | 0 (0.00) | 6 (33.33) | 4 (19.05) | 3 (12.50) |  |
|  |  | 3 | 14 (21.54) | 0 (0.00) | 5 (27.78) | 3 (14.29) | 6 (25.00) |  |
|  |  | x | 7 (10.77) | 1 (50.00) | 2 (11.11) | 4 (19.05) | 0 (0.00) |  |
|  | M(%) | 0 | 23 (35.38) | 1 (50.00) | 6 (33.33) | 9 (42.86) | 7 (29.17) | 0.0052 |
|  |  | 1 | 40 (61.54) | 0 (0.00) | 12 (66.67) | 11 (52.38) | 17 (70.83) |  |
|  |  | x | 2 (3.08) | 1 (50.00) | 0 (0.00) | 1 (4.76) | 0 (0.00) |  |
| BMI (mean (SD)) |  |  | 22.116 (3.432) | 23.985 (6.300) | 23.196 (2.839) | 20.842 (3.509) | 22.266 (3.415) | 0.1491 |
| weight loss (%) |  | <5 | 46 (73.02) | 1 (50.00) | 13 (76.47) | 16 (80.00) | 16 (66.67) | 0.6533 |
|  |  | ≥5 | 17 (26.98) | 1 (50.00) | 4 (23.53) | 4 (20.00) | 8 (33.33) |  |
| PD-1/L1 | Cachexia (%) | No | 48 (73.85) | 1 (50.00) | 15 (83.33) | 16 (76.19) | 16 (66.67) | 0.5463 |
|  |  | Yes | 17 (26.15) | 1 (50.00) | 3 (16.67) | 5 (23.81) | 8 (33.33) |  |
|  | Camrelizumab (%) | No | 50 (76.92) | 2 (100.00) | 13 (72.22) | 15 (71.43) | 20 (83.33) | 0.6288 |
|  |  | Yes | 15 (23.08) | 0 (0.00) | 5 (27.78) | 6 (28.57) | 4 (16.67) |  |
|  | Sintilimab (%) | No | 46 (70.77) | 2 (100.00) | 10 (55.56) | 15 (71.43) | 19 (79.17) | 0.3003 |
|  |  | Yes | 19 (29.23) | 0 (0.00) | 8 (44.44) | 6 (28.57) | 5 (20.83) |  |
|  | Tislelizumab (%) | No | 52 (80.00) | 1 (50.00) | 17 (94.44) | 16 (76.19) | 18 (75.00) | 0.2574 |
|  |  | Yes | 13 (20.00) | 1 (50.00) | 1 (5.56) | 5 (23.81) | 6 (25.00) |  |
|  | Pembrolizumab (%) | No | 59 (90.77) | 2 (100.00) | 17 (94.44) | 20 (95.24) | 20 (83.33) | 0.4614 |
|  |  | Yes | 6 (9.23) | 0 (0.00) | 1 (5.56) | 1 (4.76) | 4 (16.67) |  |
|  | Atezolizumab (%) | No | 60 (92.31) | 2 (100.00) | 18 (100.00) | 18 (85.71) | 22 (91.67) | 0.3969 |
|  |  | Yes | 5 (7.69) | 0 (0.00) | 0 (0.00) | 3 (14.29) | 2 (8.33) |  |
|  | Toripalimab (%) | No | 58 (89.23) | 1 (50.00) | 18 (100.00) | 20 (95.24) | 19 (79.17) | 0.0336 |
|  |  | Yes | 7 (10.77) | 1 (50.00) | 0 (0.00) | 1 (4.76) | 5 (20.83) |  |
|  | Durvalumab (%) | No | 57 (87.69) | 2 (100.00) | 16 (88.89) | 20 (95.24) | 19 (79.17) | 0.3872 |
|  |  | Yes | 8 (12.31) | 0 (0.00) | 2 (11.11) | 1 (4.76) | 5 (20.83) |  |
|  | Nivolumab (%) | No | 63 (96.92) | 2 (100.00) | 17 (94.44) | 21 (100.00) | 23 (95.83) | 0.7538 |
|  |  | Yes | 2 (3.08) | 0 (0.00) | 1 (5.56) | 0 (0.00) | 1 (4.17) |  |
|  | Cadonilimab (%) | No | 64 (98.46) | 2 (100.00) | 18 (100.00) | 20 (95.24) | 24 (100.00) | 0.5463 |
|  |  | Yes | 1 (1.54) | 0 (0.00) | 0 (0.00) | 1 (4.76) | 0 (0.00) |  |
| Location of irAEs | Liver(%) | No | 64 (98.46) | 2 (100.00) | 18 (100.00) | 21 (100.00) | 23 (95.83) | 0.6292 |
|  |  | Yes | 1 (1.54) | 0 (0.00) | 0 (0.00) | 0 (0.00) | 1 (4.17) |  |
|  | Thyroid(%) | No | 62 (95.38) | 1 (50.00) | 17 (94.44) | 21 (100.00) | 23 (95.83) | 0.0153 |
|  |  | Yes | 3 (4.62) | 1 (50.00) | 1 (5.56) | 0 (0.00) | 1 (4.17) |  |
|  | Skin(%) | No | 62 (95.38) | 1 (50.00) | 16 (88.89) | 21 (100.00) | 24 (100.00) | 0.0041 |
|  |  | Yes | 3 (4.62) | 1 (50.00) | 2 (11.11) | 0 (0.00) | 0 (0.00) |  |
|  | Lung(%) | No | 64 (98.46) | 2 (100.00) | 18 (100.00) | 21 (100.00) | 23 (95.83) | 0.6292 |
|  |  | Yes | 1 (1.54) | 0 (0.00) | 0 (0.00) | 0 (0.00) | 1 (4.17) |  |
|  | Heat(%) | No | 64 (98.46) | 2 (100.00) | 18 (100.00) | 21 (100.00) | 23 (95.83) | 0.6292 |
|  |  | Yes | 1 (1.54) | 0 (0.00) | 0 (0.00) | 0 (0.00) | 1 (4.17) |  |
| Grade of irAEs | Grade1-2 (%) | No | 58 (89.23) | 1 (50.00) | 16 (88.89) | 21 (100.00) | 20 (83.33) | 0.0855 |
|  |  | Yes | 7 (10.77) | 1 (50.00) | 2 (11.11) | 0 (0.00) | 4 (16.67) |  |
|  | Grade3-4 (%) | No | 64 (98.46) | 1 (50.00) | 18 (100.00) | 21 (100.00) | 24 (100.00) | <0.0001 |
|  |  | Yes | 1 (1.54) | 1 (50.00) | 0 (0.00) | 0 (0.00) | 0 (0.00) |  |
| Duration of PD-1/L1  (week, median [IQR]) |  |  | 18.000 [12.000, 24.000] | 37.500 [29.250, 45.750] | 19.500 [14.000, 24.000] | 18.000 [10.000, 24.000] | 15.000 [11.250, 21.000] | 0.1522 |
| Follow-up time (day, median [IQR]) |  |  | 167.000 [118.000, 287.000] | 346.000 [278.000, 414.000] | 173.000 [141.750, 332.750] | 167.000 [78.000, 272.000] | 162.000 [101.750, 239.500] | 0.353 |

CR, complete response; PR, partial response; SD^*^, stable disease; PD, progressive disease; SD, standard deviation; IQR, interquartile range.

Table S12. Factors affecting diseases progression by univariate cox regression analysis

| Factors | | Statistic | HR |
| --- | --- | --- | --- |
| Sex | Female | 10 (18.5%) |  |
|  | Male | 44 (81.5%) | 1.32 (0.53-3.29, P=.551) |
| Age | Mean ± SD | 58.2 ± 12.3 | 1.02 (0.98-1.05, P=.316) |
| T | 1 | 7 (13.0%) |  |
|  | 2 | 5 (9.3%) | 1.62 (0.36-7.31, P=.532) |
|  | 3 | 10 (18.5%) | 1.96 (0.44-8.76, P=.379) |
|  | 4 | 32 (59.3%) | 1.69 (0.45-6.34, P=.439) |
| N | 0 | 11 (20.4%) |  |
|  | 1 | 17 (31.5%) | 1.14 (0.41-3.15, P=.799) |
|  | 2 | 12 (22.2%) | 0.83 (0.21-3.37, P=.796) |
|  | 3 | 14 (25.9%) | 0.94 (0.32-2.72, P=.906) |
| M | 0 | 21 (38.9%) |  |
|  | 1 | 33 (61.1%) | 1.12 (0.49-2.53, P=.786) |
| TNM | III | 16 (29.6%) |  |
|  | IV | 38 (70.4%) | 1.37 (0.55-3.43, P=.498) |
| weight loss(%) | Mean ± SD | 0.8 ± 4.8 | 1.05 (0.96-1.14, P=.309) |
| Cachexia | No | 40 (74.1%) |  |
|  | Yes | 14 (25.9%) | 1.42 (0.64-3.17, P=.393) |
| immunofluorescence score | Mean ± SD | 0.5 ± 0.1 | 0.03 (0.00-1.57, P=.081) |
| immunofluorescence group | high | 27 (50.0%) |  |
|  | low | 27 (50.0%) | 2.39 (1.02-5.63, P=.046) |
| irAEs | No | 48 (88.9%) |  |
|  | Yes | 6 (11.1%) | 0.95 (0.32-2.81, P=.919) |
| With chemotherapy | No | 29 (53.7%) |  |
|  | Yes | 25 (46.3%) | 1.21 (0.56-2.63, P=.627) |
| With target therapy | No | 46 (85.2%) |  |
|  | Yes | 8 (14.8%) | 1.97 (0.77-5.01, P=.155) |
| With surgical | No | 49 (90.7%) |  |
|  | Yes | 5 (9.3%) | 0.37 (0.05-2.77, P=.335) |

SD, stable disease.

Table S13. REAGENT or RESOURC

| REAGENT or RESOURCE | SOURCE | IDENTFIER |
| --- | --- | --- |
| Matrigel matrix | Corning, NY, USA | Cat#356231 |
| Papain | Sigma-aldrich | Cat#P4762 |
| Collagenase IV | Sigma-aldrich | Cat#C5138 |
| DNaseI | Roche Life Science | Cat#10104159001 |
| FoxP3/Transcription Factor Staining Buffer Kit | MultiSciences Biotech | Cat#IC001 |
| Anti-mouse CD45(30-F11), PE-Cy7 | TONBO biosciences | Cat# 60-0451 |
| Anti-Mouse CD326 (Ep-CAM)(G8.8), PerCP/Cyanine5.5 | BioLegend | Cat#118220 |
| Anti-Mouse CD31(390),SB600 | eBioscience | Cat#63-0311-82 |
| Anti-Mouse CD140b (PDGFRB) (APB5), APC-eFluor™ 780 | eBioscience | Cat#47-1402-82 |
| Anti-Mouse CD140a (PDGFRA) (APA5), APC-eFluor™ 780 | eBioscience | Cat#47-1401-82 |
| Anti-Mouse Pdpn(8.1.1), APC | BioLegend | Cat#127410 |
| Anti-Mouse Dpp4(H194-112), FITC | BioLegend | Cat#137806 |
| Anti-Mouse Ltbr(3C8), PE | Invitrogen | Cat#12-5671-82 |
| MMP-9 antibody (E-11), PE | santa | Cat#sc-393859 PE |
| Anti-Mouse CD24(M1/69), EF450 | eBioscience | Cat#48-0242-82 |
| Anti-MouseCD4(RM4-5), FITC | TONBO biosciences | Cat#35-0042-U100 |
| Anti-Mouse CD8a (53-6.7), APC | TONBO biosciences | Cat#20-0081-U100 |
| Anti-Mouse CD197(4B12), PE | eBioscience | Cat#12-1971-82 |
| Goat anti-mouse IgG | BioLegend | Cat#405331 |
| Anti-Mouse ter119,PE | TONBO biosciences | Cat#50-5921-U100 |
| Anti-Mouse CD71(R17217 (RI7 217.1.4)),APC | Invitrogen | Cat#17-0711-82 |
| Anti-Mouse Pdpn | Abcam,Cambridge,MA | Cat#ab256559 |
| Anti-Mouse Ltβr | Invitrogen | Cat#PA5-102743 |
| MMP-9 antibody (E-11)，Mouse IgG2b | santa | Cat#sc-393859 |
| Anti-Mouse MIP-3 beta | santa | Cat#sc-74233 |
| Goat anti-rat Alexa Fluor 488 | Abcam,Cambridge,MA | Cat#ab150165 |
| Goat anti-rabbit Alexa Fluor 594 | Abcam,Cambridge,MA | Cat#ab150088 |
| Goat anti-mouse Alexa Fluor 647 | Abcam,Cambridge,MA | Cat#ab150119 |
| Goat anti-mouse Alexa Fluor 594 | Abcam,Cambridge,MA | Cat#ab150116 |
| Goat anti-Human IgG (H+L) | Invitrogen | Cat#A-11013 |
| DAPI | Abcam,Cambridge,MA | Cat#ab104139 |
| Anti-mouse CD3 (17A2) | BioLegend | Cat#100238 |
| Mice Mmp9 Ab ELISA Kit | MEIMIAN | Cat#MM-47001M1 |
| Mice Hmgcs2 Ab ELISA Kit | MEIMIAN | Cat#MM-46994M1 |
| Anti-mouse CD45, biotin | Invitrogen | Cat#13-0451-82 |
| MagniSort™ Streptavidin Positive Selection Beads | Invitrogen | Cat#MSPB-6003-74 |
| EasySep™ Mouse CD45 Positive Selection Kit | Stemcell,Vancouver, Canada | Cat#18945 |

Supplementary references

S1. Li Y, Li K, Zhu L, Li B, Zong D, Cai P, et al. Development of double-positive thymocytes at single-cell resolution. Genome medicine. 2021;13:49. doi:10.1186/s13073-021-00861-7

S2. Karimi MM, Guo Y, Cui X, Pallikonda HA, Horková V, Wang YF, et al. The order and logic of CD4 versus CD8 lineage choice and differentiation in mouse thymus. Nature communications. 2021;12:99. doi:10.1038/s41467-020-20306-w

S3. Ma J, Liu Y, Duan C, Wu S, Xie Y, Yang L, et al. CD226 knockout reduces the development of CD8+ T by impairing the TCR sensitivity of double-positive thymocytes. Immunology. 2023;169:83-95. doi:10.1111/imm.13612

S4. Hato L, Vizcay A, Eguren I, Pérez-Gracia JL, Rodríguez J, Gállego Pérez-Larraya J, et al. Dendritic Cells in Cancer Immunology and Immunotherapy. Cancers. 2024;16:doi:10.3390/cancers16050981

S5. Steier Z, Aylard DA, McIntyre LL, Baldwin I, Kim EJY, Lutes LK, et al. Single-cell multiomic analysis of thymocyte development reveals drivers of CD4(+) T cell and CD8(+) T cell lineage commitment. Nature immunology. 2023;24:1579-90. doi:10.1038/s41590-023-01584-0

S6. Davidson S, Efremova M, Riedel A, Mahata B, Pramanik J, Huuhtanen J, et al. Single-Cell RNA Sequencing Reveals a Dynamic Stromal Niche That Supports Tumor Growth. Cell reports. 2020;31:107628. doi:10.1016/j.celrep.2020.107628

S7. Barut GT, Kreuzer M, Bruggmann R, Summerfield A, Talker SC. Single-cell transcriptomics reveals striking heterogeneity and functional organization of dendritic and monocytic cells in the bovine mesenteric lymph node. Frontiers in immunology. 2022;13:1099357. doi:10.3389/fimmu.2022.1099357

S8. Giroux M, Denis F. CD1d-unrestricted human NKT cells release chemokines upon Fas engagement. Blood. 2005;105:703-10. doi:10.1182/blood-2004-04-1537

S9. Feuerer M, Hill JA, Mathis D, Benoist C. Foxp3+ regulatory T cells: differentiation, specification, subphenotypes. Nature immunology. 2009;10:689-95. doi:10.1038/ni.1760

S10. Stockis J, Liénart S, Colau D, Collignon A, Nishimura SL, Sheppard D, et al. Blocking immunosuppression by human Tregs in vivo with antibodies targeting integrin αVβ8. Proceedings of the National Academy of Sciences of the United States of America. 2017;114:E10161-e8. doi:10.1073/pnas.1710680114

S11. Bosteels C, Neyt K, Vanheerswynghels M, van Helden MJ, Sichien D, Debeuf N, et al. Inflammatory Type 2 cDCs Acquire Features of cDC1s and Macrophages to Orchestrate Immunity to Respiratory Virus Infection. Immunity. 2020;52:1039-56.e9. doi:10.1016/j.immuni.2020.04.005

S12. Sagar, Pokrovskii M, Herman JS, Naik S, Sock E, Zeis P, et al. Deciphering the regulatory landscape of fetal and adult γδ T-cell development at single-cell resolution. The EMBO journal. 2020;39:e104159. doi:10.15252/embj.2019104159

S13. Shen H, Gu C, Liang T, Liu H, Guo F, Liu X. Unveiling the heterogeneity of NKT cells in the liver through single cell RNA sequencing. Scientific reports. 2020;10:19453. doi:10.1038/s41598-020-76659-1

S14. Brioschi S, Wang WL, Peng V, Wang M, Shchukina I, Greenberg ZJ, et al. Heterogeneity of meningeal B cells reveals a lymphopoietic niche at the CNS borders. Science (New York, NY). 2021;373:doi:10.1126/science.abf9277

S15. Wang J, Loveless I, Adrianto I, Liu T, Subedi K, Wu X, et al. Single-cell analysis reveals differences among iNKT cells colonizing peripheral organs and identifies Klf2 as a key gene for iNKT emigration. Cell discovery. 2022;8:75. doi:10.1038/s41421-022-00432-z

S16. Wegrzyn AS, Kedzierska AE, Obojski A. Identification and classification of distinct surface markers of T regulatory cells. Frontiers in immunology. 2022;13:1055805. doi:10.3389/fimmu.2022.1055805

S17. Giampaolo S, Chiarolla CM, Knöpper K, Vaeth M, Klein M, Muhammad A, et al. NFATc1 induction by an intronic enhancer restricts NKT γδ cell formation. iScience. 2023;26:106234. doi:10.1016/j.isci.2023.106234

S18. Marrero I, Hamm DE, Davies JD. High-throughput sequencing of islet-infiltrating memory CD4+ T cells reveals a similar pattern of TCR Vβ usage in prediabetic and diabetic NOD mice. PloS one. 2013;8:e76546. doi:10.1371/journal.pone.0076546

S19. Marrero I, Aguilera C, Hamm DE, Quinn A, Kumar V. High-throughput sequencing reveals restricted TCR Vβ usage and public TCRβ clonotypes among pancreatic lymph node memory CD4(+) T cells and their involvement in autoimmune diabetes. Molecular immunology. 2016;74:82-95. doi:10.1016/j.molimm.2016.04.013

S20. Kasmani MY, Ciecko AE, Brown AK, Petrova G, Gorski J, Chen YG, et al. Autoreactive CD8 T cells in NOD mice exhibit phenotypic heterogeneity but restricted TCR gene usage. Life science alliance. 2022;5:doi:10.26508/lsa.202201503

S21. Voigt A, Bohn K, Sukumaran S, Stewart CM, Bhattacharya I, Nguyen CQ. Unique glandular ex-vivo Th1 and Th17 receptor motifs in Sjögren's syndrome patients using single-cell analysis. Clinical immunology (Orlando, Fla). 2018;192:58-67. doi:10.1016/j.clim.2018.04.009

S22. Wanchoo A, Voigt A, Sukumaran S, Stewart CM, Bhattacharya I, Nguyen CQ. Single-cell analysis reveals sexually dimorphic repertoires of Interferon-γ and IL-17A producing T cells in salivary glands of Sjögren's syndrome mice. Scientific reports. 2017;7:12512. doi:10.1038/s41598-017-12627-6

S23. Zhao Y, Nguyen P, Vogel P, Li B, Jones LL, Geiger TL. Autoimmune susceptibility imposed by public TCRβ chains. Scientific reports. 2016;6:37543. doi:10.1038/srep37543

S24. Oftedal BE, Ardesjö Lundgren B, Hamm D, Gan PY, Holdsworth SR, Hahn CN, et al. T cell receptor assessment in autoimmune disease requires access to the most adjacent immunologically active organ. Journal of autoimmunity. 2017;81:24-33. doi:10.1016/j.jaut.2017.03.002

S25. Hermansson A, Ketelhuth DF, Strodthoff D, Wurm M, Hansson EM, Nicoletti A, et al. Inhibition of T cell response to native low-density lipoprotein reduces atherosclerosis. The Journal of experimental medicine. 2010;207:1081-93. doi:10.1084/jem.20092243

S26. Nakayama M, Michels AW. Using the T Cell Receptor as a Biomarker in Type 1 Diabetes. Frontiers in immunology. 2021;12:777788. doi:10.3389/fimmu.2021.777788

S27. Okamura T, Hamaguchi M, Tominaga H, Kitagawa N, Hashimoto Y, Majima S, et al. Characterization of Peripheral Blood TCR in Patients with Type 1 Diabetes Mellitus by BD Rhapsody(TM) VDJ CDR3 Assay. Cells. 2022;11:doi:10.3390/cells11101623

S28. Nakagawa R, Muroyama R, Saeki C, Oikawa T, Kaise Y, Koike K, et al. CD4(+) T cells from patients with primary biliary cholangitis show T cell activation and differentially expressed T-cell receptor repertoires. Hepatology research : the official journal of the Japan Society of Hepatology. 2019;49:653-62. doi:10.1111/hepr.13318

S29. Hong X, Meng S, Tang D, Wang T, Ding L, Yu H, et al. Single-Cell RNA Sequencing Reveals the Expansion of Cytotoxic CD4(+) T Lymphocytes and a Landscape of Immune Cells in Primary Sjögren's Syndrome. Frontiers in immunology. 2020;11:594658. doi:10.3389/fimmu.2020.594658

S30. Nettersheim FS, Ghosheh Y, Winkels H, Kobiyama K, Durant C, Armstrong SS, et al. Single-cell transcriptomes and T cell receptors of vaccine-expanded apolipoprotein B-specific T cells. Frontiers in cardiovascular medicine. 2022;9:1076808. doi:10.3389/fcvm.2022.1076808
